# Whole-genome resequencing of the native sheep provides insights into the microevolution and identifies genes associated with reproduction traits

**DOI:** 10.1186/s12864-023-09479-y

**Published:** 2023-07-11

**Authors:** Mengting Zhu, Yonglin Yang, Hua Yang, Zongsheng Zhao, Hongmei Zhang, Hugh T. Blair, Wei Zheng, Mingyuan Wang, Chenhui Fang, Qian Yu, Huaqian Zhou, Hangdong Qi

**Affiliations:** 1 State Key Laboratory of Sheep Genetic Improvement and Healthy Production, Xinjiang Academy of Agricultural and Reclamation Science, Shihezi, China; 2 College of Animal Science and Technology, Shihezi University, Shihezi, Xinjiang China; 3 First Affiliated Hospital, School of Medical College, Shihezi University, Shihezi, Xinjiang China; 4 Institute Veterinary, Animal & Biomedical Sciences, Massey University, Auckland, Palmerston North, New Zealand

**Keywords:** Microevolution, Selection signature, Whole-genome resequencing, GWAS, Reproductive trait, Sheep

## Abstract

**Background:**

Sheep genomes undergo numerous genes losses, gains and mutation that generates genome variability among breeds of the same species after long time natural and artificial selection. However, the microevolution of native sheep in northwest China remains elusive. Our aim was to compare the genomes and relevant reproductive traits of four sheep breeds from different climatic environments, to unveil the selection challenges that this species cope with, and the microevolutionary differences in sheep genomes. Here, we resequenced the genomes of 4 representative sheep breeds in northwest China, including Kazakh sheep and Duolang sheep of native breeds, and Hu sheep and Suffolk sheep of exotic breeds with different reproductive characteristics.

**Results:**

We found that these four breeds had a similar expansion experience from ~ 10,000 to 1,000,000 years ago. In the past 10,000 years, the selection intensity of the four breeds was inconsistent, resulting in differences in reproductive traits. We explored the sheep variome and selection signatures by *F*_ST_ and θπ. The genomic regions containing genes associated with different reproductive traits that may be potential targets for breeding and selection were detected. Furthermore, non-synonymous mutations in a set of plausible candidate genes and significant differences in their allele frequency distributions across breeds with different reproductive characteristics were found. We identified *PAK1, CYP19A1* and *PER1* as a likely causal gene for seasonal reproduction in native sheep through qPCR, Western blot and ELISA analyses. Also, the haplotype frequencies of 3 tested gene regions related to reproduction were significantly different among four sheep breeds.

**Conclusions:**

Our results provide insights into the microevolution of native sheep and valuable genomic information for identifying genes associated with important reproductive traits in sheep.

**Supplementary Information:**

The online version contains supplementary material available at 10.1186/s12864-023-09479-y.

## Background

Sheep (*Ovis aries*) was one of the most important economic animals in the world. It played an important role in agriculture, economy and culture in the early civilization of mankind. All these make sheep one of the most successful animals domesticated by humans in the early Neolithic Age [1]. In the poem ‘Book of Songs, Xiaoya, Wu Yang’ who says there are no sheep? Three hundred flocks of sheep" vividly depicts the scene of sheep flourishing more than 3,000 years ago [2]. Prof. Li 's team used genome, archaeological records and ethnic historical materials to find that the formation of sheep native breeds was related to the nomadic migration route about 3000–5000 years ago, and analyzed the migration, diffusion and variety formation of sheep in China [3, 4]. Based on this, it can be found that sheep not only helped the survival of early humans but also contributed to solving the world 's poverty [5]. On the other hand, it is well known that the complex genetic background of Xinjiang sheep and the wide variation in the performance of economically important traits have provided researchers with more genetic material [6]. Furthermore, studies of ancient introgression in native animals have indicated that infiltration of important economic traits is an important resource of genetic variation in natural populations and may contribute to improvement during domestication [7].

As we all know, Xinjiang has unique conditions for the development of sheep farming, that there are 13 local breeds and 4 cultivate breeds in Xinjiang. Through the long-term operation of animal husbandry, Xinjiang people had carefully cultivated many excellent sheep breeds, which generally had the advantages of strong adaptability, prolificacy, and strong resistance to disease and environmental stresses. For example, Kazakh sheep have meat and fat production performance and are highly adaptable to the ecological conditions of the production area [8]. The Duolang sheep are an excellent meat and fat sheep breed in Xinjiang, with a larger body, more meat production, excellent characteristics of perennial estrus, high reproduction rate and stable heredity [6]. Hu sheep have excellent traits such as early maturity, perennial estrus and high fertility [9]. Suffolk sheep have the characteristics of rapid growth and production of muscular carcasses and well adaptability [10]. Wang et al. found that Duolang sheep, Hu sheep and Altay sheep were close to native Chinese sheep and far away from the wild sheep breeds [11]. Since the selection and breeding of native sheep in Xinjiang was characterized by seasonal breeding, studying their microevolutionary relationships could resolve the genetic basis of reproductive performance differences at the genomic level [8]. It has been reported that Kazakh sheep and Hu sheep belong to Mongolian sheep, but there was no direct research evidence [9, 12]. Although previous studies have focused on sheep domestication, there is no research on the evolution of representative native sheep in Xinjiang. Most of the Xinjiang native breeds of sheep currently kept are seasonally bred, and seasonal breeding of sheep is the main cause of inefficiency in the entire Xinjiang sheep farming industry and the main bottleneck of efficient modern sheep farming [8]. Therefore, understanding the microevolution process of native sheep was of great significance for analyzing the genetic basis of the study on conservation and adaptive evolution of germplasm resources in sheep.

Many studies have analyzed the genetic evolution of domestic animal genomes adapted to extreme environment. The team of Ji et al. conducted genome-wide resequencing of 77 Chinese native sheep breeds from different geographical environments, compared the sheep genomes in extreme environments, such as plateaus and plains, arid deserts and humid areas, and found a series of new genes related to the adaptability of sheep to extreme environments [13]. Similarly, Li and his team uncovered a hitherto unknown contribution of *PAPSS2* to high-altitude adaptability in Tibetan goats, following interspecific introgression and natural selection [14]. Liu et al. analyzed the microevolutionary relationship among 3 Chinese short fat-tailed sheep breeds by whole genome resequencing, and found *TSHR* and *PRL* gene were played an important role in reproduction [15]. Xinjiang's unique topography of "three mountains sandwiched by two basins" has formed three unique ecosystems of mountains, oases and deserts, which belongs to the temperate monsoon climate and have obvious seasonal changes [16, 17]. Yang et al. reported transcriptome sequencing of spleen tissue from the native and exotic sheep breeds were performed via Illumina high-throughput sequencing technology, which highlighted that the adaptability of native sheep breeds to adversity was stronger than that of exotic breeds and screened *LCP2, HP* and *CSF1R* genes that may be important genes affecting sheep adaptability [10, 18]. However, the genetic basis of microevolutionary differences in these sheep breeds and the marker genes controlling sheep reproductive traits and their regulatory mechanisms are still unclear.

Therefore, in order to clarify the relationship between Kazakh sheep and Duolang sheep with significant characteristics in the local area, as well as the differences and microevolutionary relationships in reproductive performance and key genes of reproductive traits between the offspring of the exotic high-fertility Hu sheep and the offspring of the Suffolk sheep with outstanding growth and development performance. We compared the genetic evolution relationship of native Kazakh sheep, Duolang sheep, Hu sheep and Suffolk sheep, the microevolution process was discussed and the candidate genes related to reproductive traits were screened. To find the variation and selection signals related to microevolution that adapt to the environment and affect the reproductive phenotype, and construct a reference group for further studying the regulation mechanism of reproductive traits. Finally, explore the variation and selection signals related to microevolution that adapt to the environment and affect the reproductive phenotype, and construct a reference group for further studying the regulation mechanism of reproductive traits, which provided the basis for the creation of new germplasm of native sheep.

## Result

### Sequencing, mapping, and SNP/indel detection

**Fig. 1:**
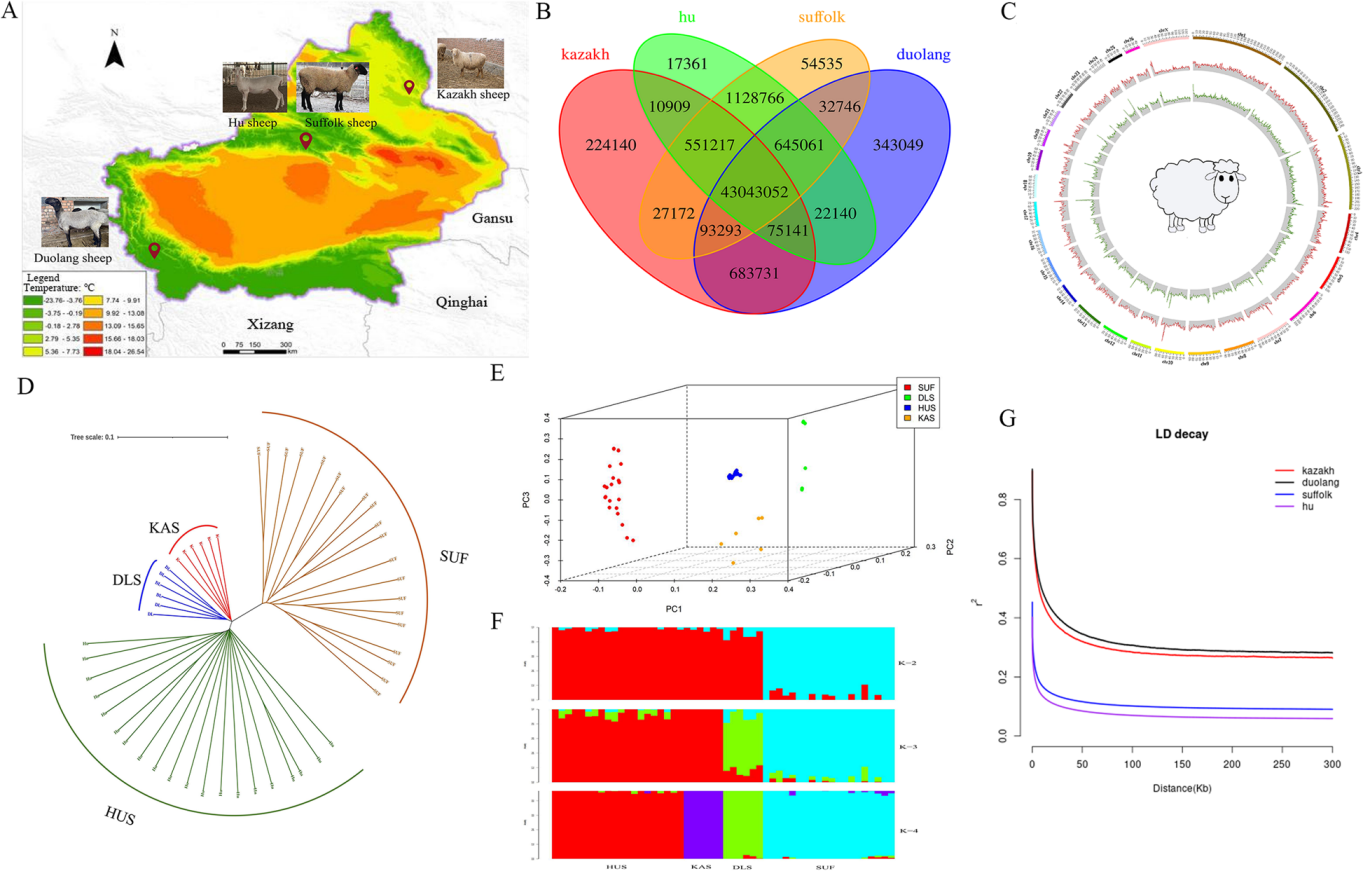
Geographic distribution of native sheep breeds. **A** Geographical distribution of four sheep breeds, this figure has been modified from Geographical Information Monitoring Cloud Platform (http://www.dsac.cn/). **B** Venn diagram of SNPs distribution in sheep population. **C** SNPs and InDels density distribution circle. **D** Neighbor-joining tree of four genetic groups based on the IBS genetic distances. **E** PCA principal component analysis. **F** Population structure of 52 individuals using ADMIXTURE with k = 2, 3, 4. **G** LD decay of four sheep breeds

High-depth whole-genome resequencing of the 52 samples of Xinjiang native sheep breeds (Fig. 1A and Supplementary Data 2) generated a total of 2808.75 Gb, with an average depth of 17 × per individual and an average genome coverage of 98.74%. The GC content was 43.23% – 46.62%, mean Q20 >  = 95% mean Q30 >  = 89% and the Mapping rate after alignment with the reference genome reached more than 99% (Supplementary Data 2). There was no significant difference in sequence coverage among individuals from the four groups (Kruskal–Wallis, *P* > 0.05). We obtained a total of 639,170,248 high-quality SNPs and 96,314,524 high-quality InDels after mapping with SAMtools, respectively (Supplementary Data 3). According to the result, nonsynonymous/synonymous ratio of SNPs for four population is 0.90, 0.90, 0.70 and 0.72, respectively. According to the distribution in chromosomes, we can find that the InDels results are consistent with the distribution trend of SNPs, and both show the most distribution in the intergenic region, both reaching more than 60% (Supplementary Data 3). After filtering, a final set of 181,278,251 high-quality SNPs were obtained (44,802,053 SNPs were identified in Kazakh sheep population, 45,039,737 SNPs in Duolang sheep population, 45,676,298 SNPs in Hu sheep population, and 45,760,163 SNPs in Suffolk sheep population), among which, 43,043,052 common SNPs were obtained from the four breeds, while 224,140, 343,049, 17,361 and 54,535 special SNPs in the Kazakh sheep, Duolang sheep, Hu sheep and Suffolk sheep, respectively (Fig. 1B). To get a clearer picture of the distribution of sheep chromosomal SNPs and InDels, a window of 100 Kb was set, and density maps of SNPs and InDels were counted for each window (Fig. 1C). Obviously, it can be seen that SNPs and InDels have good homogeneity.

### Demographic history and microevolution analysis

**Fig. 2:**
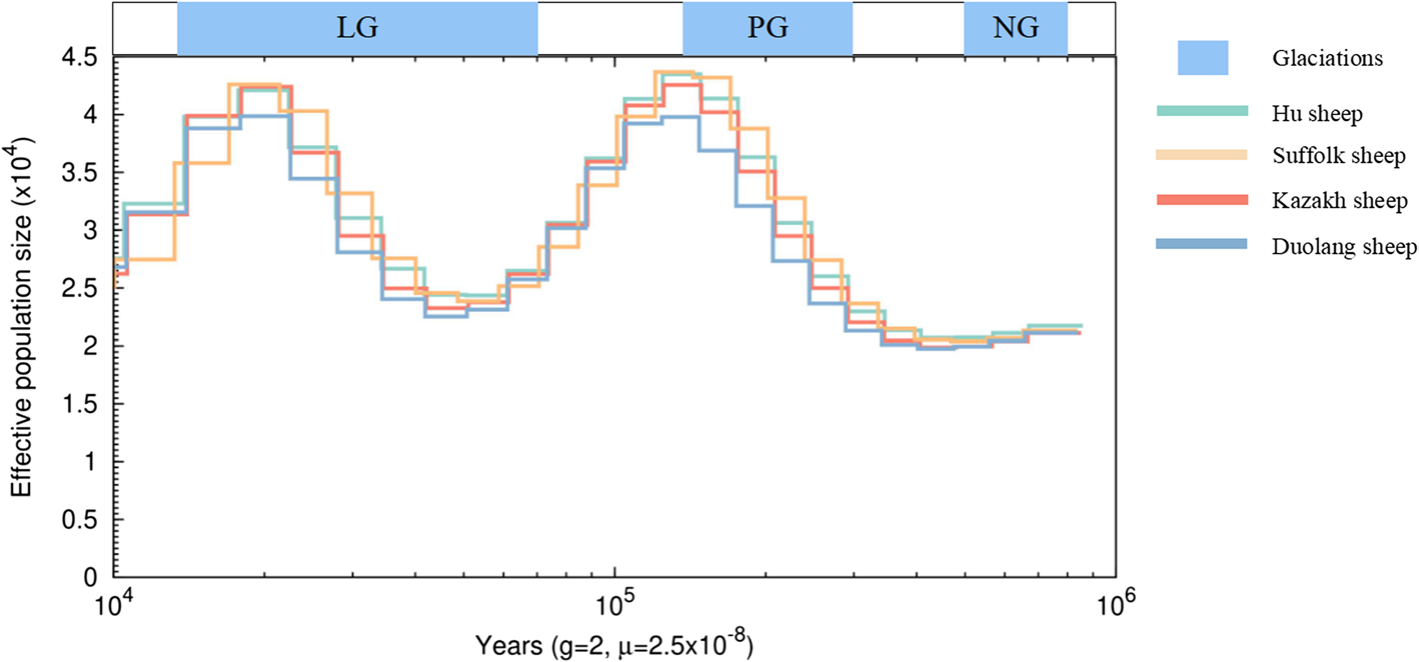
Pairwise sequential Markovian coalescent (PSMC) analysis results. Evolutionary effective population size of four Xinjiang native sheep breeds over the last 10^6^ years. LG: last glaciation. PG: Penultimate glaciation. NG: Naynayxungla glaciation

Pairwise sequentially Markovian coalescent (PSMC) analysis revealed concordant demographic trajectories and microevolution for native sheep breed, with two expansions and two contractions in Ne during the last one million years (Fig. 2). The first bottleneck occurred ~ 0.58 Mya, during which the population size was the lowest, and is consistent with the Naynayxungla glaciation (0.78–0.50 Mya). The first expansion occurred as the climate entered the glacial period, and the population size increased and peaked occurred ~ 0.18–0.14 Mya, during the Penultimate glaciation (0.3–0.13 Mya). About 70,000 years ago, the effective population size was reduced here, which was the last glacial period (0.13–0.07 Mya), because it was caused by the large glacier coverage. The second expansion in the last glacial period (26.5–19 Ka) due to the reduction of glacier coverage, ecological improvement, grassland area expanded, about 18,000 years ago sheep effective population size gradually increased. Obviously, during this period, the microevolution trends of the four sheep breeds were similar and the number of Suffolk sheep was the least.

### Population genetic structure, linkage disequilibrium

The NJ tree revealed that the four sheep groups were separated into four branches. Obviously, Kazakh sheep, Duolang sheep and Hu sheep were close, but far away from Suffolk sheep (Fig. 1D). For another, PCA clustering analysis provided additional corroborating evidence for these groups. It also indicated a good consistency of the selected samples (Fig. 1E). In the clustering analysis, when K = 4, the four groups could be distinguished (Fig. 1F). Linkage disequilibrium (LD, measured as r^2^) decreased to half of its maximum value at 11.1 kb and 15.2 kb in Kazakh and Duolang but at 3 kb and 2.35 kb in Suffolk and Hu breeds, respectively. Clearly, Hu population decay fastest, Duolang population decay slowest, and the rank of LD for the four sheep breeds was Hu sheep > Suffolk sheep > Kazakh sheep > Duolang sheep (Fig. 1G).

### Selective imprints and microevolution of reproduction trait during domestication and breeding

To explore the genome-wide selection signatures influenced by microevolution in artificial selection, we compared the four sheep populations and analyzed for selection signals using a 100 kb sliding window size and step length of 50 kb across genome, a combination of both *F*_ST_ (Supplementary Fig. 1 and Supplementary Data 4) and nucleotide diversity (π) methods (Supplementary Fig. 2 and Supplementary Data 5) to scan for selection signals on autosomes and screen sheep for genomic regions. Top 5% *F*_ST_ and π ratio were selected as the threshold to draw the selected loci on autosomes between two populations. Of these sweeps identified, we focused on genes involved in seasonal reproduction, and annotated functional genes with high credibility (Supplementary Data 9), including some previously reported (e.g., *GDP9, CYP19A1, 3β-HSD, PLCB1, MED12L, PER1, FSHR, WNT4, NOTCH2, RXRG, LHR, HTR5A, PIK3R3, LOC101118189, LOC101122280,* et al.). And the *PAK1, GNAQ, TSHR* genes were also associated with reproductive traits by selective signature analysis. They were mainly enriched in ovarian steroidogenesis, steroid biosynthesis, cAMP signaling pathway, MAPK signaling pathway, calcium signaling pathway, circadian rhythm, GnRH signaling pathway, TGF-beta signaling pathway and other signaling pathways, which may be involved in the regulation of hormone secretion (Supplementary Data 7 and Supplementary Figs. 4 and 5).

**Fig. 3:**
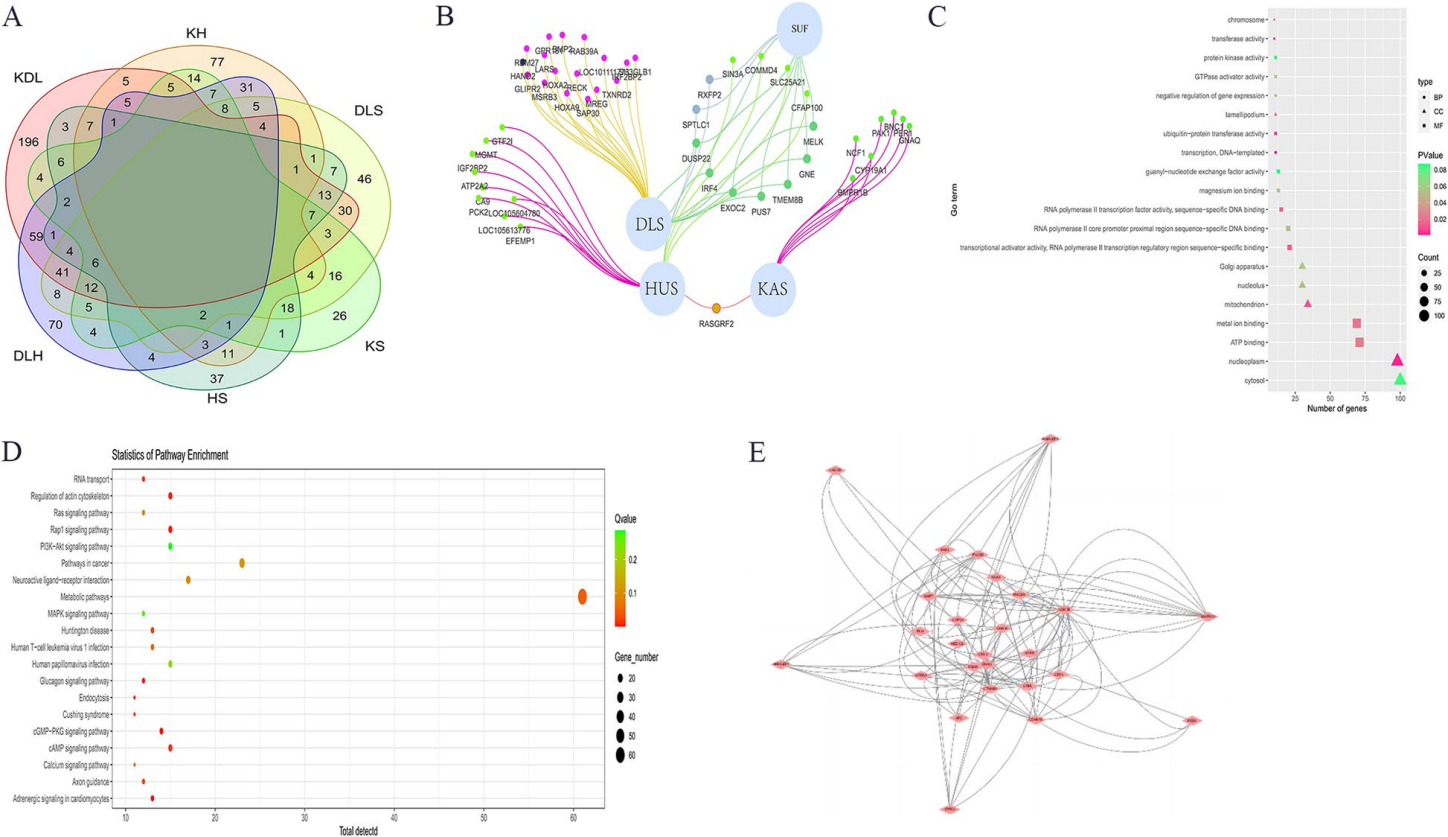
Specific selection signals of 4 sheep breeds (**A**) Venn diagram of common and specific selected region of four sheep breeds; (**B**) Venn network of special gene; (**C**) Top 20 GO results (**D**) Top 20 KEGG results; (**E**) PPI results

The specific selection signals of the four sheep breeds during domestication and artificial selection were shown in Fig. 3, which shows that the Kazakh sheep had 9 specific selected regions with a length of 0.88 Mb (0.33‰ of the genome), contained 8 protein-coding genes; the Duolang sheep had 28 specific selected regions with a length of 2.73 Mb (1.03‰ of the genome), contained 24 protein-coding genes; the Hu sheep had 19 specific selected regions with a length of 1.86 Mb (0.37‰ of the genome), contained 16 protein-coding genes; and the Suffolk sheep had 11 specific selected regions with a length of 1.07 Mb (0.70‰ of the genome), contained 9 protein-coding genes (Fig. 3A and B). GO and KEGG analysis of candidate genes in the selected region revealed that they were mainly enriched in signaling pathways such as Ras signaling pathway, MAPK signaling pathway, cushing syndrome, calcium signaling pathway and nucleoplasm, ATP binding, metal ion binding, and DNA templat processes (Fig. 3C and D).

**Fig. 4:**
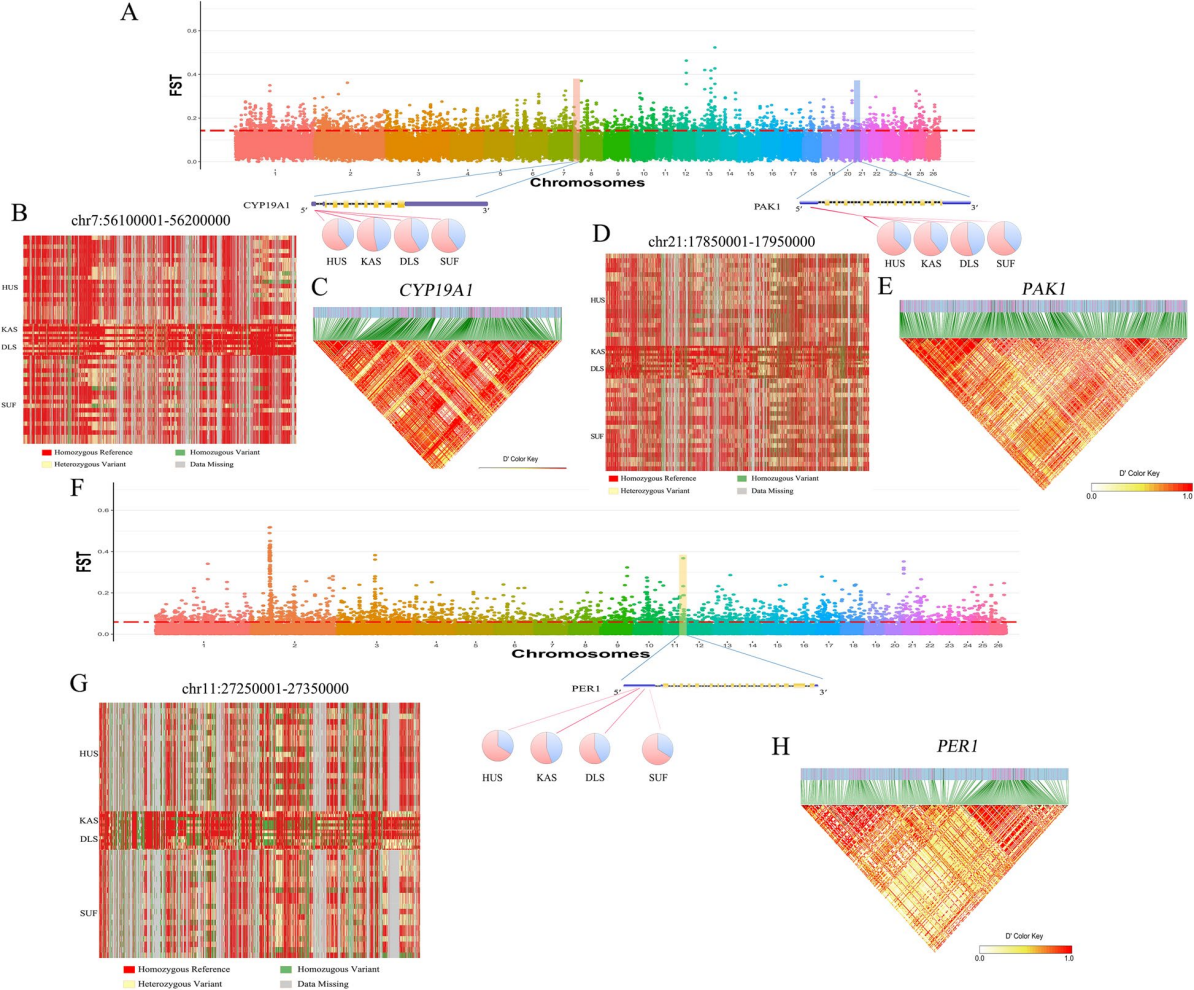
Genome-wide annotations during sheep of seasonal reproduction. **A** Selection signals in Kazakh sheep and Hu sheep. **B**, **D**, **G** Haplotype diversity of a local region of *CYP19A1* (chromosome 7: 56100001–56200000), *PAK1* (chromosome 21: 17850001–17950000) and *PER1* (chromosome 11: 27250001–27350000). **C**, **E**, **H** Linkage disequilibrium analysis of SNPs in *CYP19A1*, *PAK1* and *PER1* region. The pie charts represent the spectrum of allele frequencies at the non-synonymous loci of the focused genes *PAK1, PER1* and *CYP19A1* in sheep breeds. The type of variant allele is indicated in pink, whereas the reference allele in blue

Through the combined analysis of resequencing results and GWAS results, we identified *PAK1, PER1* and *CYP19A1* were associated with reproduction traits on chromosome 21, chromosome 11 and chromosome 9, respectively, was identified. The genotype pattern of *PAK1*, *PER1* and *CYP19A1* showed significant differences between low prolific sheep (KAS and SUF) and high prolific sheep (HUS and DLS), and linkage disequilibrium analysis indicated strong linkage of SNPs in these region (Fig. 4, Supplementary Fig. 3 and Supplementary Data 6). To further understand the important candidate genes associated with reproductive traits in sheep, the genes of the above grouping of intersecting signaling pathways *PAK1, PER1, CYP19A1, FSHR, LHR, 3β-HSD, PLCB1, MED12L, HTR5A, GNAQ, HTR5A, PIK3R3, GSK3B, PIK3R2, MAPK10, RAPGEF1, PLN, ITPR2, GNA14*, etc., and their interactions were analyzed using string online software, and the results are shown in Fig. 3E.

### Genome-wide association analysis of sheep reproductive traits

**Fig. 5:**
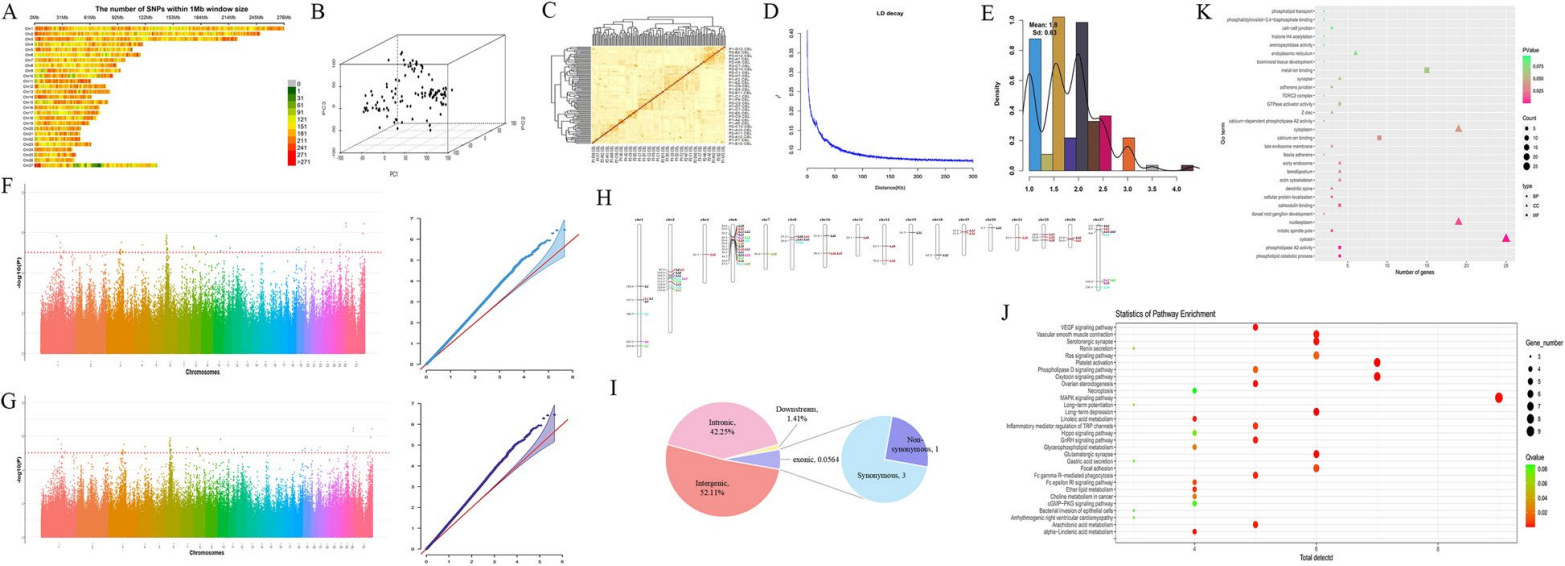
GWAS analysis. **A** Distribution and density of SNP on Sheep chromosomes (**B**) PCA analysis. **C** Kinship. **D** LD Decay. **E** Phenotypic frequency distribution of average litter size in prolific Suffolk sheep. **F** GLM Manhattan and QQ plot. **G** Farm CPU Manhattan and QQ plot. **H** Location of physical map of SNPs. **I** Distribution of SNPs. **J** GO analysis. **K** KEGG pathway analysis. Note: GLM and Farm CPU models manhattan threshold line -log10 (*p*) = 5

Affymetrix Ovis 600 K chip was used to genotype 115 prolific Suffolk sheep (Fig. 5 E and Table 1). A density map was drawn based on the position of the SNP on the chromosome (Fig. 5A). PCA maps was used as PC1, PC2 and PC3 to construct a genetic relationship G using SNP loci (Fig. 5B). For another, the population structure is relatively stable, the selected sample. The consistency is good, and there is no outlier sample, which could be used for follow-up experiments (Fig. 5C). Besides, when the physical position increases, the LD coefficient (r^2^) shows a gradual downward trend, and when the physical position is 100 Kb, r^2^ tends to be balanced (Fig. 5D). GLM and Farm CPU methods were used to GWAS analysis on the average litter size traits of prolific Suffolk sheep (Fig. 5F-H). According to the Fig. 5I, 70 important SNPs, including 61 on the autosome and 9 on the X chromosome were scanned (Supplementary Data 10). Further annotation found that they were mainly distributed in the Intergenic region (52.11%), followed by the Intronic region (42.25%) (Fig. 5J). Further alignment to the Animal QTL database according to the physical location of SNPs, 4 QTLs for Reproductive seasonality (ASREP) and 6 QTLs for Testes weight (TESTWT) (Supplementary Data 11).

**Table 1:** Phenotypic descriptive statistics

Trait	Max	Min	Mean	Median	Std.dev	SE.mean	Coef.var
Average litter size	4.33	1	1.81	1.75	0.63	0.62	0.35

Affx-281083833 locus on chr 20 was annotated to *BMPR1B* gene, Affx-281083833 locus was annotated to *BMP5* and other star genes related to litter size traits in sheep. Interestingly, Affx-281158636 on chr 21 was annotated to *PAK1* gene, Affx-281088123 on chr11 was annotated to *PER1* gene, Affx-281177422 on chr7 was annotated to *CYP19A1* and other genes related to sheep reproductive traits, which were consistent with the genes related to sheep reproductive traits screened by resequencing (Table 2).

**Table 2:** SNPs and genes associated with reproductive traits

SNPs						QTLs		
SNP	CHR	POS/bp	p-value	gene	ra/ea	QTL start/cM	QTL end/cM	Traits
Affx-281177422	7	59,903,215	6.25E-06	*CYP19A1*	G/A	73.73	77.32	Average daily gain QTL#13972
Affx-281158636	21	25,535,979	1.21E-06	*PAK1*	T/A	16.8	59.2	Average daily gain QTL#13946
				*TBRG1*				
Affx-281088123	11	25,120,385	9.06E-06	*PER1*	T/C	3.7	64.9	Body weight QTL#14297
				*CNTROB*				
Affx-281162367	6	30,103,189	7.72E-06	*HPGDS*	T/C	3.5	120.7	Body weight QTL#14284
Affx-280811112	6	29,382,188	1.34E-06	*BMPR1B*	T/C	45	76.1	Body weight QTL#14261
Affx-280754995	1	245,315,235	1.67E-05	*RASA2*	G/A	228	316.8	Reproductive seasonality #QTL16603
Affx-281010617	25	23,781,151	6.85E-06	*CTNNA3*	C/T	0	76	Testes weight QTL#12925
Affx-281126803	19	13,246,866	8.30E-06	*CTNNB1*	G/A	0	27.7	Reproductive seasonality QTL#16612
Affx-281012499	19	13,494,863	5.60E-06	*CTNNB1*	G/A	0	27.7	Reproductive seasonality QTL#16612
Affx-281083833	20	4,718,474	6.85E-06	*BMP5*	C/T	0	28	Testes weight QTL#12927
Affx-281068952	1	186,677,951	8.06E-06	*MYLK*	C/T	0	28	Testes weight QTL#12927

Traits all traits significantly associated with the locus; *CHR* the chromosome; *POS* the position of the SNP on the chromosome (according to the OAR3.1 assembly); *ra/ea* reference and effectivealleles

Further, GO and KEGG enrichment analysis showed that candidate genes were enriched in important economic traits such as reproductive traits, immune adaptability, growth and development traits. Notably, the KEGG pathway results of serotonergic synapse, ovarian steroidogenesis, cAMP signaling pathway, GnRH signaling pathway, MAPK signaling pathway and other signaling pathways related to reproductive traits were consistent with the enrichment pathways of candidate genes significantly related to sheep reproductive traits screened by resequencing (Fig. 5K and Supplementary Data 12). These results are also consistent with the breeding process of prolific Suffolk sheep, which further confirms the hypothesis that the litter size of sheep is regulated by multiple genes.

### Effects of *PAK1*, *CYP19A1* and *PER1* on reproductive endocrine

**Fig. 6:**
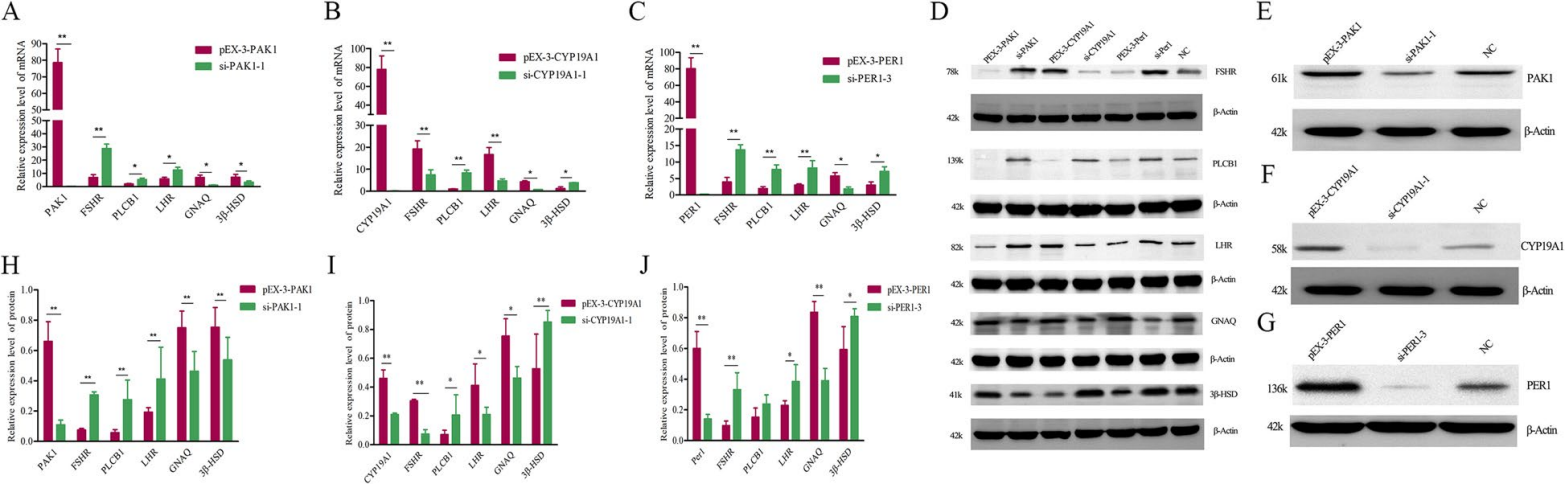
Effects of *PAK1* (**A**, **H**), *CYP19A1* (**B**, **I**), *PER1* (**C**, **J**) genes on mRNA and protein levels of seasonal reproduction-relative genes (**D**, **E**, **F**, **G**).* represented a significant difference (*P* < 0.05), **represented a extremely significant difference (*P* < 0.01)

**Fig. 7:**
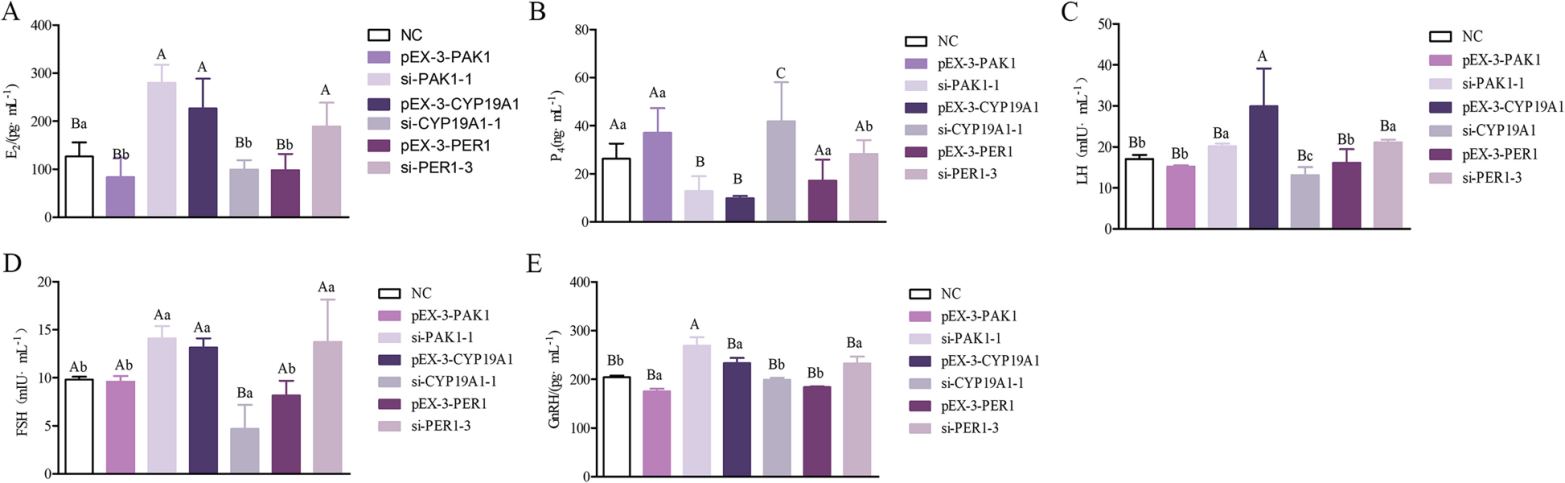
Expression results of E_2_ (**A**), P_4_ (**B**), LH (**C**), FSH (**D**), GnRH (**E**). Different lowercase letters indicate the difference is significant (*P* < 0.05), and different uppercase letters indicate the difference is extremely significant (*P* < 0.01)

Based on the results of resequencing and GWAS, the major genes *CYP19A1, PER1* and *PAK1*, which affect reproductive traits in sheep, were selected to investigate their regulatory mechanisms. According to Fig. 6, the pEX-3-PAK1, pEX-3-CYP19A1, pEX-3-PER1, si-PAK1-1, si-CYP19A1-1 and si-PER1-3 were transfected into sheep GCs cells, respectively. Then, the untreated group was used as the control. The effects of *PAK1, CYP19A1* and *PER1* on the differential expression of genes related to reproductive trait were detected by qPCR and WB, respectively, to further study their regulatory relationship. The expression of *PAK1* gene in pEX-3-PAK1 group was significantly higher than that in si-PAK1-1 group (*P* < 0.01). The *PAK1* upstream gene *FSHR* was significantly lower than the si-PAK1-1 group (*P* < 0.01). The expression of downstream genes *PLCB1* and *LHR* were significantly lower in the overexpression group than in the interference group (*P* < 0.05), while the downstream genes *GNAQ* and *3β-HSD* were significantly higher in the pEX-3-PAK1 group than in the si-PAK1-1 group (*P* < 0.05). Compared with NC, down-regulation of *PAK1* increased E_2_, LH, FSH and GnRH secretion, up-regulation of *PAK1* promoted P_4_ secretion (Fig. 7). The expression of *CYP19A1* gene in pEX-3-CYP19A1 group was significantly higher than that in si-CYP19A1-1 group (*P* < 0.01), while the expression of *FSHR* and *LHR*, upstream genes of *CYP19A1*, was significantly higher than that in si-CYP19A1-1 group (*P* < 0.01). The downstream gene *PLCB1* was significantly lower in the overexpression group than in the interference group (*P* < 0.01). *GNAQ* also showed that the overexpression group was significantly higher than the interference group (*P* < 0.05). *3β-HSD* in pEX-3-CYP19A1 group was significantly lower than that in si-CYP19A1-1 group (*P* < 0.05) (Fig. 6). In contrast to *PAK1*, overexpression of *CYP19A1* increased E_2_, LH, FSH and GnRH secretion, whereas downregulation of *CYP19A1* was promoted P_4_, which opposited to the role of the *PAK1* (Fig. 7). The expression of *PER1* gene in pPEX-3-PER1 group was significantly lower than that in si-PER1-3 group (*P* < 0.01). The *PER1* upstream genes *FSHR* and *LHR* and downstream genes *PLCB1* were significantly lower than si-PER1-3 group (*P* < 0.01). The expression of the downstream gene *GNAQ* was significantly higher in the overexpression group than in the interference group (*P* < 0.05). The relative expression of downstream gene *3β-HSD* in pEX-3-PER1 group was significantly lower than that in si-PER1-3 group (*P* < 0.05) (Fig. 6). Down-regulation of *PER1* increased E_2_, LH, FSH and GnRH secretion, down-regulation of *PER1* increased P_4_ (Fig. 7).

### Effects of *PAK1*, *CYP19A1* and *PER1* genes on proliferation and apoptosis of GCs cells

**Fig. 8:**
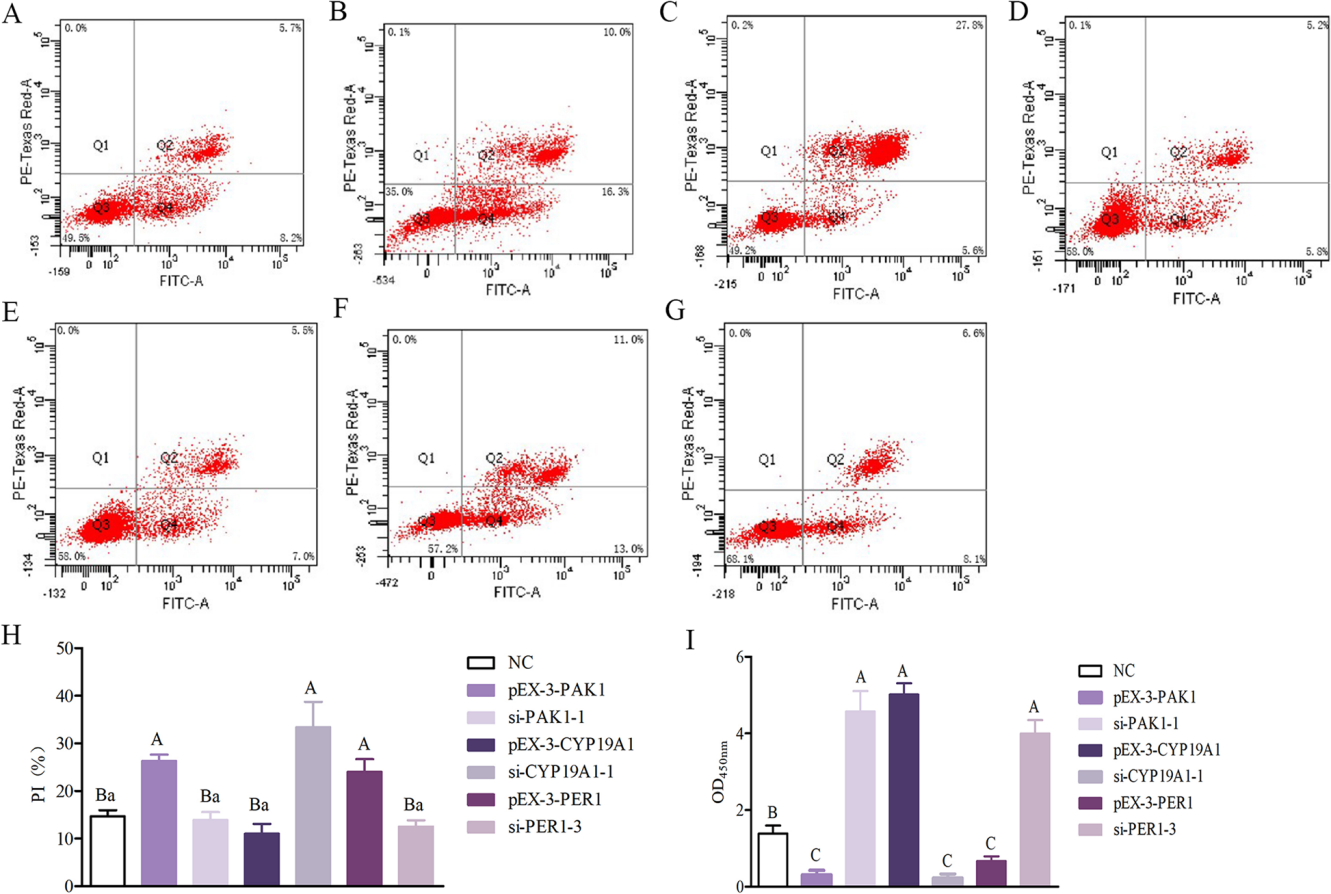
Effects of *PAK1*, *CYP19A1* and *PER1* Genes on Apoptosis and Proliferation of GCs Cells (**A**) si-PAK1-1; (**B**) pEX-3-PAK1; (**C**) si-CYP19A1-1; (**D**) pEX-3-CYP19A1; (**E**) si-PER1-3; (**F**) pEX-3-PER1; (**G**) NC; (**H**) PI statistics; (**I**) Effects of *PAK1, CYP19A1* and *PER1* Genes on Proliferation of GCs Cells

From Fig. 8, after transfection of *PAK1*, *CYP19A1*, *PER1* gene overexpression recombinant plasmids and interference fragments, CCK-8 and flow cytometry were used to detect the effect of each recombinant vector on the proliferation of sheep GCs cells at 48 h after transfection, respectively. Compared with the NC group, the si-PAK1-1 group could promote the proliferation of GCs cells (*P* < 0.01) and inhibit apoptosis, while the pEX-3-PAK1 group inhibited the proliferation of GCs (*P* < 0.01) and promoted apoptosis, which was the same as the down-regulation of *PAK1* gene to promote the proliferation of GCs cells. In contrast, compared with the NC group, the pEX-3-CYP19A1 group could promote the proliferation of GCs cells (*P* < 0.01), while the si-CYP19A1-1 group inhibited the proliferation of GCs cells (*P* < 0.01). It can be seen that up-regulation of *CYP19A1* can inhibit apoptosis, while down-regulation of this gene can increase the apoptosis rate. Compared with NC group, pEX-3-PER1 group could significantly inhibit the proliferation of GCs cells (*P* < 0.01), but could promote the apoptosis of GCs cells, while down-regulation of *PER1* gene could promote the proliferation of GCs cells (*P* < 0.01) and inhibit the apoptosis.

## Discussion

Our selection analyses revealed that Kazakh sheep, Duolang sheep, Hu sheep and Suffolk sheep contain many common selection signatures and unique selection signatures represented its characters among their genomes. We found that four sheep breeds experienced two expansions and two bottlenecks 10,000 years ago, which closed to those of the mouflon sheep [13]. Additionally, during this period, it was similar to the historical effective population evolution trend of giant panda [19] and monkey [20]. Further, we observed that Hu sheep had the fastest decay rate, while Duolang sheep had the slowest decay rate, which may be due to the fact that Xinjiang herdsmen have strengthened the breeding of local sheep breeds, especially for reproductive traits and meat traits. It was consistent with the results reported by Li et al. [21]. Further, through the analysis of microevolution, the regions of selection identified in four native breeds harbor different protein-coding genes with important biological functions, including roles in development, reproduction, growth and adaptability, which was consistent with the results reported by Liu [15].

Reproductive performance was one of the important economic traits in sheep breeding industry. Litter size was the largest production trait in important economic traits, and its contribution rated to sheep industry can reach 70–90% [15, 22]. It is reported that the economic benefits of double lambs are more than 1.6 times that of single lambs [23]. Seasonal estrus was the main bottleneck restricting the high prolificacy of sheep [24]. Therefore, deciphering the genetic basis of artificial selection and animal domestication is an active research area. The availability of whole-genome sequences provides an opportunity to study this at the gene mutation level. Such genomic studies have been implemented in other domestic animals [25, 26], and also in sheep [16]. In this study, the genetic variation of important economic traits, especially reproductive traits, was excavated.

Furthermore, through selective imprints the *PAK1, CYP19A1, 3β-HSD, PLCB1, PER1, FSHR, WNT4, GNAQ, NOTCH2* and other genes related to sheep reproductive traits were mainly enriched in ovarian steroidogenesis, steroid biosynthesis, cAMP signaling pathway, calcium signaling pathway, circadian rhythm, GnRH signaling pathway and other signaling pathways. In addition to the discovery of *CYP19A1, PER1, 3β-HSD, FSHR*, and *LHR* related to sheep reproductive traits reported in previous studies [27–29], *PAK1* and *GNAQ* genes were also excavated, of which *GNAQ* gene and previous studies of this research group found that *GNAQ* may be related to seasonal estrus in sheep [30, 31]. Besides, the results showed that *EFNA5, FGFR3, NCAPG, FGF22, THBS2,* genes related to sheep growth and development traits were enriched in PI3K-AKT, AMPK and other signaling pathways. Zhu et al. [32, 33] found that PI3K-AKT signaling pathway may be involved in poultry early embryonic development and early breast and leg muscle development. Further, we used the prolific Suffolk breeding group as a resource group to carry out GWAS research. Its reproduction rate was as high as 185% and the reproduction rate of prolific Suffolk ewes with FecB BB genotype could even reach 225.0% [10]. A total of 70 SNPs were significantly associated with the average litter size and 156 candidate genes were screened by GWAS. Interestingly, the signal pathways enriched by genes related to seasonal reproduction were partially consistent with the resequencing results. The annotated *BMPR1B* and *BMP5* genes are consistent with the marker genes related to litter size and ovulation rate in sheep reported previously [34, 35]. The results of this study are also consistent with the production and breeding process of prolific Suffolk sheep, which could also confirm the authenticity of the results in the article.

**Fig. 9:**
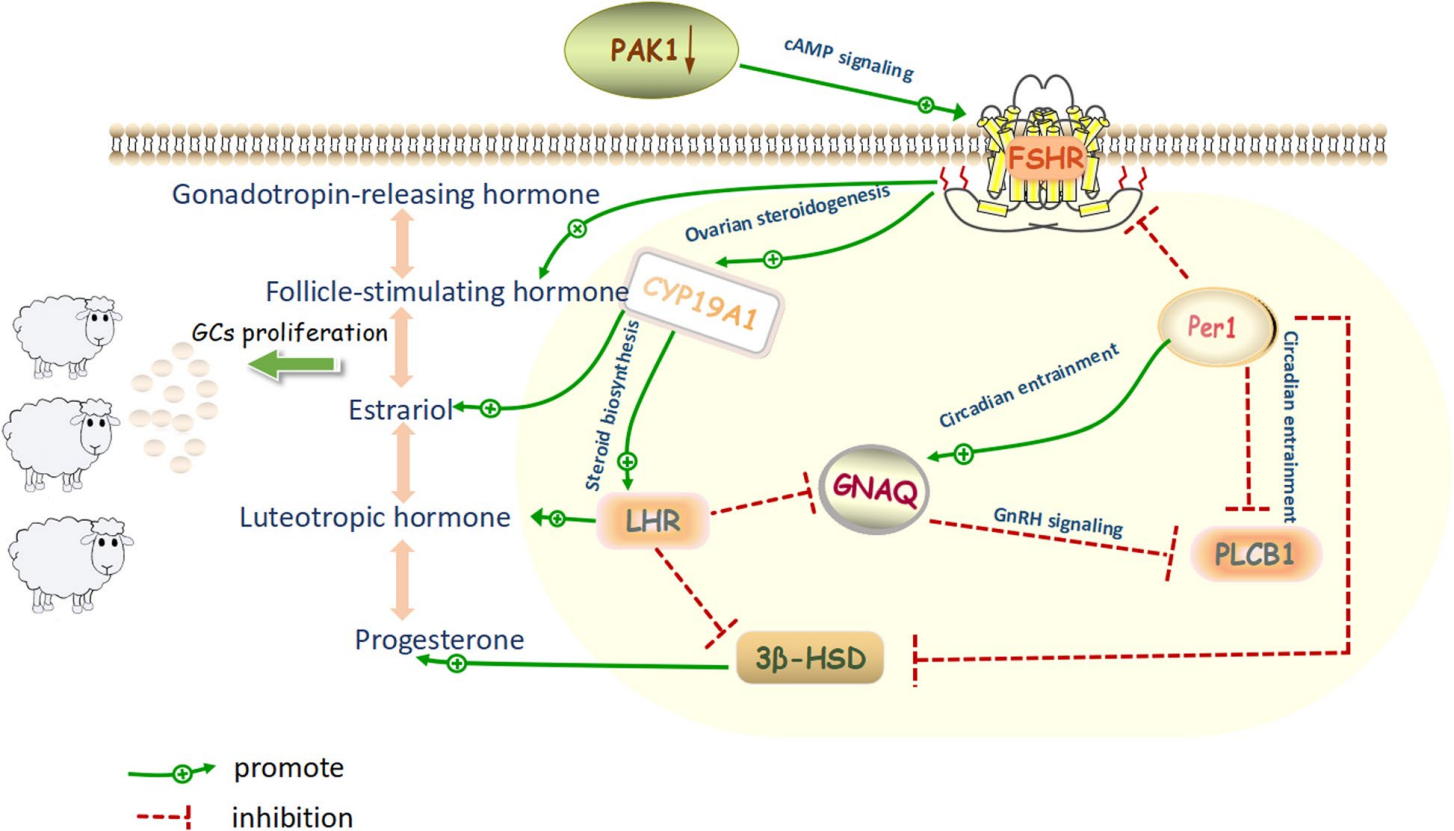
Regulation of *PAK1, CYP19A1, PER1* may be involved in sheep reproduction

In summary, *PAK1, CYP19A1* and *PER1* genes may play an important role in the seasonal reproduction of sheep, and further analyze the regulatory process of *PAK1, CYP19A1* and *PER1* on seasonal reproduction of sheep. Reproduction of sheep involves the regulation of the hypothalamic-pituitary-ovarian axis by affecting the active state of the ovary under positive and negative feedback [36–38]. The result showed that down-regulation of *PAK1* gene may promote the binding of FSH to its receptor *FSHR* through cAMP signaling pathway, promote the up-regulation of *FSHR*, up-regulate its upstream genes *LHR* and *PLCB1*, and also promote the activity of aromatase, resulting in the up-regulation of *CYP19A1*, down-regulation of downstream genes *PER1*, *GNAQ* and *3β-HSD* expression, thus affecting the secretion of E_2_, P_4_, FSH, LH and GnRH, promoting the proliferation of ovarian GCs cells, thus affecting the reproductive performance of sheep (Fig. 9). This result was consistent with the report of Zhang et al. in goat GCs [39]. In addition, Zhao et al. demonstrated in a mouse model that *PAK1* mediates a negative regulation of E_2_ in the non-classical ERα pathway in the reproductive axis, and also confirmed that *PAK1* inhibits E_2_ secretion in this study, thereby inhibiting follicular development and ovulation, thereby regulating sheep reproduction [40]. E_2_ and low concentration of P_4_ could play a synergistic role in promoting follicular development and ovulation process [27, 41, 42], and then regulated the estrus and litter size traits of sheep, which was similar to the results of Pilorz et al. in female animals [43]. In summary, the regulatory mechanism of *PAK1*, *CYP19A1* and *PER1* in sheep ovarian GCs cells may regulate the seasonal reproduction of sheep through cAMP signaling pathway, ovarian steroidogenesis, calcium signaling pathway, GnRH signaling pathway and Steroid biosynthesis signaling pathway was analyzed, which provided a basis for the analysis of molecular regulation mechanism of perennial estrus and high fecundity of sheep.

## Conclusion

In the study, the genetic differentiation of four sheep breeds during domestication and artificial selection was revealed from the whole genome level, and the molecular mechanism of *PAK1*, *CYP19A1* and *PER1* targeting GCs cells to regulate seasonal reproduction of sheep was analyzed. This will underpin more-accurate identification of high prolific gene variants in the near future and facilitate novel breeding strategies, like genome selection having important research significance for the development of new native sheep breeds and improvement of sheep breeding.

## Materials and methods

### Sample collection and sequencing

The samples for genome resequencing were from 4 Xinjiang native sheep breeds, including 6 Kazakh sheep, 6 Duolang sheep, 20 Hu sheep and 20 Suffolk sheep, the same age, similar weight, and healthy disease-free were selected. More specifically, 52 Xinjiang native sheep individuals represent various geographicorigins, morphological characteristics, and production traits (Supplementary Data 1). DNA samples were collected from 123 prolific Suffolk sheep ewes with similar age, body weight, and normal reproductive function in the sheep breeding farm of Xinjiang Academy of Agricultural and Reclamation Sciences for GWAS analysis. Genomic DNA was extracted following the standard phenol–chloroform extraction procedure [44]. For genome resequencing, at least 0.5 μg of genomic DNA from each sample was used to construct a library with an insert size of ~ 350 bp fragments shared by Bioruptor Pico sonicator, then paired-end sequencing libraries were constructed according to the manufacturer’s instructions (Illumina, San Diego, USA) and sequenced at ~ 20 × coverage on the Illumina HiSeq2500 Sequencer (Illumina Inc.) by Tianjin Compass Biotechnology Co., Ltd. (Tianjin, China).

### Reads mapping, variant calling and annotation

To obtain high-quality data, we removed reads containing adapters, reads with ≥ 10% unidentified nucleotides (N), and remove low-quality reads (> 50% of the read bases with a Phred quality score (i.e., Q-score) <  = 5). A total of 6,537.553 Gb of high-quality paired-end reads were generated with an average quality of 96.0% for Q20 and 91.0% for Q30. Sequencing reads were aligned to the sheep reference genome (Oar_v4.0, ftp://ftp.ncbi.nlm.nih.gov/genomes/all/GCF/000/298/735/GCF_000298735.2_Oar_v4.0/GCF_000298735.2_Oar_v4.0_genomic.fna.gz) using BWA (V0.7.12) with default parameters (mem -t 4 -k 32 -M) [45]. Subsequent processing, including duplicate removal was performed using SAMtools and PICARD (http://picard.sourceforge.net) [45, 46]. Mapping results were then converted into the BAM format and sorted using SAMtools (v1.2). On an average, 99.03% of the reads were mapped, resulting in a final average sequencing coverage of 20 × per individual. After alignment, the raw SNP/InDel sets are called by SAMtools with the parameters as ‘-q 1 -C 50 -m 2 -F 0.002 -d 1000’, and the output was further filtered using VCFtools (v.0.1.13). Moreover, to exclude SNP callings errors, variant sites with QD < 2.0, MQ < 20, FS > 60.0 were discarded. The process of indel calling was the same as described for the SNPs. Finally, SNV/InDel variations were annotated using the ANNOVAR v.2013–06-21, and the UCSC known genes were used for gene and region annotations [45, 47, 48]. The VCF file was obtained by Haplotyper and GVCFtyper, and the data were filtered by PLINK software.

Prolific Suffolk sheep had become an important germplasm resource for the study of reproduction and other traits due to their high fertility and good meat performance, and their clear genetic structure. Therefore, for GWAS analysis, Affymetrix Ovis 600 K chip containing 605,043 loci was used to genotype the 123 prolific Suffolk sheep ewes samples. After filtering the fastq file, the BWA software was used to the sheep reference genome, version Oar_3.1 (ftp://ftp.ncbi.nlm.nih.gov/genomes/all/GCF/000/298/735/GCF_000298735.1_Oar_v3.1/GCF_000298735.1_Oar_v3.1_genomic.gff.gz). The VCF file was obtained by Haplotyper and GVCFtyper, and the data were filtered by PLINK software. The loci with MAF less than 0.05, the loci with markers less than 1e-5 deviating from Hardy–Weinberg equilibrium (–maf 0.05 -hwe 1e-5), and the loci with deletion rate more than 10% were excluded (–geno 0.10). After quality control, 2,015,850 high-quality SNPs were obtained for post-analysis. Finally, SNP variations were annotated using the ANNOVAR v.2013–06-21, and the UCSC known genes were used for gene and region annotations [45, 47, 48].

### Estimates of effective population size and microevolution analysis

We used the PSMC method [49] to estimate changes in the effective population size and microevolution of sheep over the past 1 million years. PSMC analysis was performed on 52 native sheep individuals. The parameters were set as follows: -N30 -t15 -r5 -p ‘4 + 25*2 + 4 + 6. An average mutation rate (μ) of 2.5 × 10–8 per base per generation and a generation time (g) of 2 years were used for the study [5]. The different SNP marker distance bins for r^2^ analysis were used to obtain different estimates of Ne at t = 1/2c generations ago.

### Population genetic struction analysis

In order to understand the relationships between different geographic populations and same breed with different conservation actions, principal component analysis (PCA) of the 52 samples was conducted by EIGENSOFT [50]. Based on neighbor-joining (NJ) method, phylogenetic tree was constructed by PHYLIP [51], and displayed by Newick Utilities, The Interactive Tree of Life (ITOL) tool (https://itol.embl.de/) was used to display the topological structure. In addition, population structure was then inferred using the software ADMIXTURE (v1.3.0) to quantify genome-wide admixture among four native sheep breeds populations [52]. The number of genetic clusters K ranged from 2 to 4 with 10,000 maximum iterations. Linkage disequilibrium analyses were carried out with PopLDdecay (https://github.com/BGI-shenzhen/PopLDdecay). To mitigate the possible effect of LD, we implemented LD pruning using the parameter -indep-pairwise (50 10 0.2) in PLINK v.1.07 [53]. The average r^2^ values were calculated for each length of distance and the whole-genome LD was averaged across all chromosomes. The LD decay plot was depicted against the length of distance using the R script (http://www.r-project.org).

### Analysis of genome-wide selective sweep regions

Nucleotide diversity (π) and global *F*_ST_ were calculated using the vcftools v.0.1.14, with a sliding window approach (100-kb windows with 50-kb step length) [21]. The significance threshold was set to the top 5% for *F*_ST_ and π ratio [54]. Then, the windows with top 5% simultaneously considered as candidate selective regions under strong selective sweep and subsequently examined for potential candidate genes. Finally, Gene Ontology (GO) and KEGG pathways were retrieved for these genes, then analysis was performed in DAVID (https://david.ncifcrf.gov) and GO database (http://geneontology.org/) [55, 56].

### GWAS

Association analyses of average litter size was performed using GLM and FarmCPU models in rMVP package of R script [57]. PCA of the 115 prolific Suffolk sheep samples was conducted by EIGENSOFT. To avoid potential false positives in multiple comparisons, the whole-genome significance threshold was adjusted via the Bonferroni test [58]. The significantly associated SNPs threshold was set to 0.05 / effective SNPs (-log10 (p) ≥ 5.0), which was considered to be an important gene and SNPs for litter size traits in sheep. Furthermore, the quantile–quantile (Q-Q) plots of the GLM and FarmCPU for average litter size was implemented in R Bioconductor. The models used in the analysis are as follows:

$$\mathrm{GLM}\;\mathrm{model}:\;\mathrm y=\mathrm{Xa}+\mathrm{Zb}+\mathrm e$$

$$\mathrm{Farm}\;\mathrm{CPU}\;\mathrm{model}:\;{\mathrm y}_{\mathrm p}={\mathrm M}_{\mathrm p1}{\mathrm b}_1+{\mathrm M}_{\mathrm p2}{\mathrm b}_2+\cdots+{\mathrm M}_{\mathrm{pn}}{\mathrm b}_{\mathrm n}+{\mathrm S}_{\mathrm{pq}}{\mathrm d}_{\mathrm q}+{\mathrm e}_{\mathrm i}$$

Among them, y represented the phenotypic value of the average litter size trait of sheep, y_p_ was the observed value of the p^th^ individual trait, μ was the population mean of the average litter size, X was the fixed effect matrix, a was the fixed effect vector, Z was the SNPs typing vector, b was the SNPs marker vector, u was the random effect, e was the random residual, M_p1_,…, M_pn_, was the association site of n genotypes added to the model. In the first iteration, this part was empty, b_1_,…, b_n_ was the effect value corresponding to the association site, and S_pq_ was the corresponding q genotype of the p^th^ individual. d_q_ was the effect value of S_pq_, and e_i_ is the residual vector.

### QTLs and genes annotation of significant SNPs

QTL mapping was performed using the Animal QTLdb (https://www.animalgenome.org/cgi-bin/QTLdb/index) database for the selected significantly associated SNPs. In this paper, we used the sub-method to locate the QTLs of significant SNPs to further explore the genetic mechanism of SNPs affecting sheep reproductive traits. The significant SNPs were mapped to the sheep reference genome (Oar_3.1) on the NCBI website for significant site annotation. GO and KEGG pathways were retrieved for these genes, then analysis was performed in DAVID (https://david.ncifcrf.gov) and GO database (http://geneontology.org/).

### Primer design for overexpression of *PAK1*, *CYP19A1*, *PER1* gene and qPCR primer design

The primers used in this study were designed with reference to the sheep *PAK1* (Gene ID: XM_015093177.3), *PER1* (Gene ID: XM_042230393.1), *CYP19A1* (Gene ID: XM_027968636.2), *FSHR* (Gene ID: XM_027961511.2), *PLCB1* (Gene ID: XM_015095900.3), *3β-HSD* (Gene ID: NM_001318077.1), *GNAQ* (Gene ID: XM_012179252.1), *LHR* (Gene ID: XM_042253635.1), mRNA sequences published in GenBank. The qPCR primers were designed with Primer Premier 5.0. The *PAK1, CYP19A1* and *PER1* gene coding sequence (CDS) amplification primers included an upstream SalI/NotI, and NheI/NotI endonuclease site (Supplementary table 13). *β-actin* was used as the internal reference gene. The primers were synthesized by Sangon Biotech (Shanghai, China).

### Effects of *PAK1*, *CYP19A1*, *PER1* on reproductive endocrine

The successfully constructed overexpression and siRNA recombinant vectors of *PAK1, PER1* and *CYP19A1* were selected for subsequent experiments. The cell culture medium was collected 48 h after pEX-3-PAK1, si-PAK1-1, pEX-3CYP19A1, si-CYP19A1-1, pEX-3-PER1, si-PER1-3 group transfection. E_2_, P_4_, FSH, LH and GnRH secretion levels were measured using an ELISA Kit (BIM, USA) according to the manufacturer’s instructions.

### Effects of *PAK1*, *CYP19A1*, *PER1* genes on proliferation and apoptosis of GCs cells

GCs cells were plated in 96-well plates. The groups were as follows: NC, pEX-3-PAK1, si-PAK1-1, pEX-3CYP19A1, si-CYP19A1-1, pEX-3-PER1, si-PER1-3, five replicates in each group. After transfection for 48 h, 100μL of new medium and 10μL of CCK-8 reagent were added to the culture medium, and the cells were cultured at 37 °C for 3 h. The absorbance (OD) at 450 nm was detected by microplate reader, and the effects of *PAK1, CYP19A1* and *PER1* genes on cell proliferation were analyzed. On the other hand, GCs cells were plated in 6-well plates. After transfection for 48 h, Annexin V-FITC / PI double staining apoptosis detection kit was used (Multi Science, Hangzhou, China), the medium was discarded and the cells were washed with pre-cooled PBS. The cells were digested with trypsin without 0.25% EDTA and transferred to a 15 mL centrifuge tube, centrifuged at 1000 rpm for 10 min, the supernatant was washed with pre-cooled PBS, centrifuged at 1000 rpm for 10 min, the supernatant was added 500 μL 1 × Binding Buffer to resuspend the cells, 5 μL Annexin V-FITC and 10 μL PI were added to each tube. After gentle vortex mixing, incubation at room temperature away from light for 5 min, then directly using flow cytometry to detect apoptosis.

### Expression of *PAK1*, *CYP19A1* and *PER1* gene and reproduction-related genes

cDNA from the NC, pEX-3-PAK1, si-PAK1-1, pEX-3CYP19A1, si-CYP19A1-1, pEX-3-PER1, si-PER1-3 groups, respectively, were used as templates. The *FSHR*, *PLCB1*, *GNAQ*, *LHR*, *3β-HSD*, mRNA expression levels in each group were detected with qPCR. *β-actin* was used as the internal reference gene. qPCR was performed using a LightCycler 96 Real-Time qPCR System (Roche, Chicago, IL, USA). The 20-μL reaction mixture contained 1.0 μL cDNA, 10 μL 2 × SYBR Premix Ex Taq, 2 μL primers (1 μL each forward and reverse primer), and 7 μL water. The reactions were performed at 95 °C for 5 min, followed by 50 cycles at 95 °C for 30 s and 59 °C for 30 s. All reactions were performed in triplicate, and relative mRNA quantification was performed using the comparative threshold cycle (2^−ΔΔCt^) method [58]. The ΔCt values for the genes of interest were calculated using the Ct values [Ct (test) − Ct (reference)]. All experiments were performed at least in triplicate.

Protein from the NC, pEX-3-PAK1, si-PAK1-1, pEX-3CYP19A1, si-CYP19A1-1, pEX-3-PER1, si-PER1-3 groups, respectively, were used as templates. The protein extract was mixed with an equal amount of sample buffer and then separated on 10% sodium dodecyl sulfatepolyacrylamide gel electrophoresis (SDS-PAGE) gels (20 μg per lane). Subsequent electrophoresis, 80 V 20 min, 120 V 60 min, then transferred to PVDF membrane (100 mA, 2 h), and then placed in blocking solution for blocking (5% skim milk TBST solution) incubated at 37 °C for 2 h. Immunodetection was carried out with the rabbit monoclonal anti-PAK1 (dilution 1:1000; Affinity, China), rabbit monoclonal anti-CYP19A1 (dilution 1:1000; Affinity, China), rabbit monoclonal anti-PER1 (dilution 1:1000; Affinity, China), rabbit monoclonal anti-GNAQ (dilution 1:1000; Abcam, UK), rabbit monoclonal anti-PLCB1 (dilution 1:1000; Affinity, China), rabbit monoclonal anti-FSHR (dilution 1:1000; bioss, China), rabbit monoclonal anti-LHR (dilution 1:1000; Novus, USA), mouset monoclonal anti-3β-HSD (dilution 1:1000; Thermofisher, USA), Rabbit anti-beta Actin antibody (dilution 1:1,000; Abcam, UK), Goat Anti-Rabbit IgG H&L (dilution 1:10,000; Abcam, UK), Goat Anti-Mouse IgG H&L (dilution 1:10,000; Abcam, UK). Densitometric analysis was performed using Quantity One software (Bio-Rad Laboratories, Inc). The relative expression level of each protein was represented by the optical density ratio of the target band to the β-actin band.

### Statistical analysis

Each experiment was performed three times. The protein results of Western blot were analyzed using Image J software to analyze the gray value. The data are expressed as the mean ± standard error (SE); all statistical analysis were performed with SPSS 20.0 (IBM Corp., Armonk, NY, USA). The capital letters are expressed at the 0.01 level, and the difference was extremely significant; the lowercase letters indicate a significant difference at the 0.05 level.

## Supplementary Information

**Additional file 1.**

**Additional file 2.**

**Additional file 3.**

## Data Availability

The datasets generated or analyzed curing the current study are available in the Genome Sequence Archive National Genomics Data Center, Beijing Institute of Genomics (China National Center for Bioinformation) Chinese Academy of Sciences, under accession number PRJCA016288 and GVM000520.

## Abbreviations

ELISA: Enzyme linked immunosorbent assay
FSH: Follicle stimulating hormone
*F*_ST_: Fixation Index
GCs: Granulosa cells
GnRH: Gonadotropin-releasing hormone
*GNAQ*: G protein subunit alpha q
GWAS: Genome-wide association study
LH: Luteinizing hormone
P_4_: Progesterone
*PLCB1*: Phospholipase C beta 1
*β-actin*: Actin beta
qPCR: Real-time fluorescent quantitative PCR
QTL: Quantitative trait locus
RNAi: RNA interference
WB: Western Blot
