# Supplementary material for: Whole-genome resequencing of the native sheep provides insights into the microevolution and identifies genes associated with reproduction traits
Source: BMC Genomics. 2023 Jul 11;24:392. doi: 10.1186/s12864-023-09479-y (PMC10334551; doi:10.1186/s12864-023-09479-y)
Supplement: Supplementary file 2 — Additional file 2. [file 12864_2023_9479_MOESM2_ESM.docx]

Supplementary Methods

Isolation, culture and identification of sheep primary GCs cells (supplementary materials)

Isolation, culture of sheep primary GCs cells

Primary isolation and culture of primary GCs cells in sheep according to the method of Lin et al [1,2] in our team. The follicular fluid was taken with a 1 mL syringe and added to the DMEM medium containing a penicillin-streptomycin mixtur (DMED: (penicillin and streptomycin)=90%:1%). After centrifugation at 1000 rpm for 10 min, it was transferred to a 25 cm2 breathable cell culture flask, the supernatant was discarded, and an appropriate amount of DMEM complete medium (DMED: serum: (penicillin and streptomycin)=90%:10%:1%) was added to blow the cells to completely suspend the cell precipitation and placed at 37 ℃ in 5% CO_2_ for culture; the medium was changed every 48 h. Since then, the liquid was changed every 48 h.

Identification of sheep primary GCs cells

Previous studies have found that FSHR is specifically expressed in GCs cells, so it is identified by cell immunofluorescence and qPCR [3]. Immunofluorescence identification of FSHR expression. GCs cells cultured for 72 h were washed with PBS 3 times, 3 min each time. Fixed with 4 % paraformaldehyde for 10 min, and then washed with PBS 3 times, 3 min each time; add 0.5 % TritonX-100 for 15min, rinse with PBS for 5 times, 3min each time, block with 1 % BSA for 30min, then discard the liquid, add 1 % BSA diluted rabbit monoclonal anti-FSHR (1:200; bioss, China), 4 ℃ refrigerator overnight, rinse with PBS for 3 times, 5min each time. The Alexa Goat Anti-Rabbit IgG H&L (1:200; Thermofisher, USA) diluted with 1 % BSA was added in the dark, incubated in an incubator at 37 °C for 1h, and then washed with PBS for 3 times, 5min each time. DAPI was added for nuclear staining for 3 min and photographed directly with a fluorescence inverted microscope.

Additionally, because of the specific expression of FSHR in GCs, primers were designed according to the CDS region of FSHR gene published by NCBI. Total RNA was extracted from GCs cells cultured to 72 h, and reverse transcribed into cDNA. The qPCR system: 2 × SYBR Premix Ex Taq 10μL, PCR Forward Primer 1μL, PCR Reverse Primer 1μL, cDNA 1μL, ddH2O 7μL, Total 20μL. The reactions were performed at 95°C for 5 min, followed by 45 cycles of 95°C for 30 s, 60°C for 30 s, and 72°C for 30 s, 95°C for 15 s, 65°C for 60 s, 95°C for 15 s, 65°C for 15 s, and 37°C for 30s.

Construction of pEX-3-PAK1, pEX-3-CYP19A1, pEX-3-PER1, siRNA-PAK1, siRNA-CYP19A1, siRNA-PER1 vectors

Sheep GCs cDNA was used as the template; the PAK1, CYP19A1 and PER1 CDS region was amplified by PCR. The 25-μL reaction mixture contained 12.5 μL 2× Ex Taq PCR Master mix, 2 μL primers (1 μL each forward and reverse primer), 1 μL cDNA, and 9.5 μL water. The reactions were performed at 95°C for 5 min, followed by 35 cycles of 94°C for 30 s, 59°C for 30 s, and 72°C for 1 min, 72°C for 10 min, and 4°C for 2 h. Using an agarose gel recovery kit (CWBIO, Beijing, China), the target band was gel-recovered, ligated, and transformed into Escherichia coli DH5α competent cells, spread on Luria Bertani (LB) solid medium containing Ampicillin (Amp) antibiotics, and cultured overnight at 37°C. The next day, a single colony was selected and placed in LB liquid medium, and cultured at 37°C with shaking for 4-6 h. Subsequently, PCR verification was performed, and the bacterial cultures containing the target fragment were sequenced by Sangon Biotech. The bacterial solution was verified by DNA sequencing and expanded in vitro, the plasmid was extracted, digested with double enzymes, and ligated and transformed with pEX-3 expression vector. Then, the solution was spread on LB solid medium, and the sequencing of bacterial cultures with the target fragment was verified by Sangon Biotech.

According to the coding region sequence of sheep PAK1, CYP19A1 and PER1 gene published by NCBI, the RNAi fragments were designed by using the online websites GE Dharmacon ( https://horizondiscovery.com/en/ordering-and-calculation-tools/sidesign-center?rdr=true&LangType=2052&pageid=17179928204) ), thermofisher ( https://rnaidesigner.thermofisher.com/rnaiexpress/setOption.do?designO) ) and DSIR ( http://biodev.extra.cea.fr/DSIR/DSIR.html), ) according to the principle of RNA interference fragment design. Three RNAi fragments of rach genes were selected and sent to GenePharma., Ltd. for synthesis. The siRNA interference fragment information is shown in Supplementary table13.

Transfection of PAK1, CYP19A1 and PER1 overexpression vector and RNAi vector

The GCs cells were digested after the cells were about 80% confluent. The cells were starved with Opti-MEM™ medium and transfected the following day. To obtain effective transfection efficiency, the optimal ratio of transfection was determined as 1:1.5 via pre-experimental plasmid transfection. Then, the pEX-3-PAK1, pEX-3-CYP19A1, pEX-3-PER1 recombinant plasmid were transfected into three groups of cells, respectively: (1) pEX-3-PAK1 group: 1) NC, 2) pEX-3-PAK1, 3) pEX-3; (2) pEX-3-CYP19A1 group: 1) NC, 2) pEX-3-CYP19A1, 3) pEX-3; (3) pEX-3-PER1 group: 1) NC, 2) pEX-3-PER1, 3) pEX-3. Additionally, the each RNAi recombinant plasmid was transfected into four groups of cells: (1) si-PAK1 group: 1) NC, 2) si-PAK1-1, 3) si-PAK1-2, 4)si-PAK1-3; (2) si-CYP19A1 group: 1) NC, 2) si-CYP19A-1, 3) si-CYP19A-2, 4)si-CYP19A-3; (3) si-PER1 group: 1) NC, 2) si-PER1-1, 3) si-PER1-2, 4)si-PER1-3. Three parallel wells were set for each group, and transfection was performed according to the instructions for Lipofectamine 3000 (Invitrogen,Thermo Fisher Scientific, USA).

Supplementary Results

Isolation, culture and identification of sheep ovarian granulosa cells

The newly isolated GCs from sheep follicles were small round granules or oval granules. In the incubator by 37 °C, 5 % CO_2_ culture 12h began to adhere to the wall, which was spindle. After 24h basically completed the adherent process, spindle began to become larger, triangular or irregular shape. After 48 h, the cell density increased and the outline was clearer. After 72 h, the round rate was about 85 % (Fig. 1A). Since FSHR is specifically expressed in GCs cells, it is not expressed in other follicular cells. Therefore, the isolated and cultured primary GCs cells were identified by immunofluorescence and qPCR. It can be seen from the figure that red fluorescence was visible in the positive cells that expressed FSHR (Fig. 1B). The nuclei of GCs cells stained with DAPI emitted blue (Fig. 1C). Nuclear and cytoplasmic staining completely coincided (Fig. 1D). The CDS sequence of 190 bp was obtained by qPCR reaction of GCs cell-specific gene FSHR, indicating that the culture and identification of sheep GCs cells were successful, and the purity of the cells was higher than 95 % (Fig. 1E).


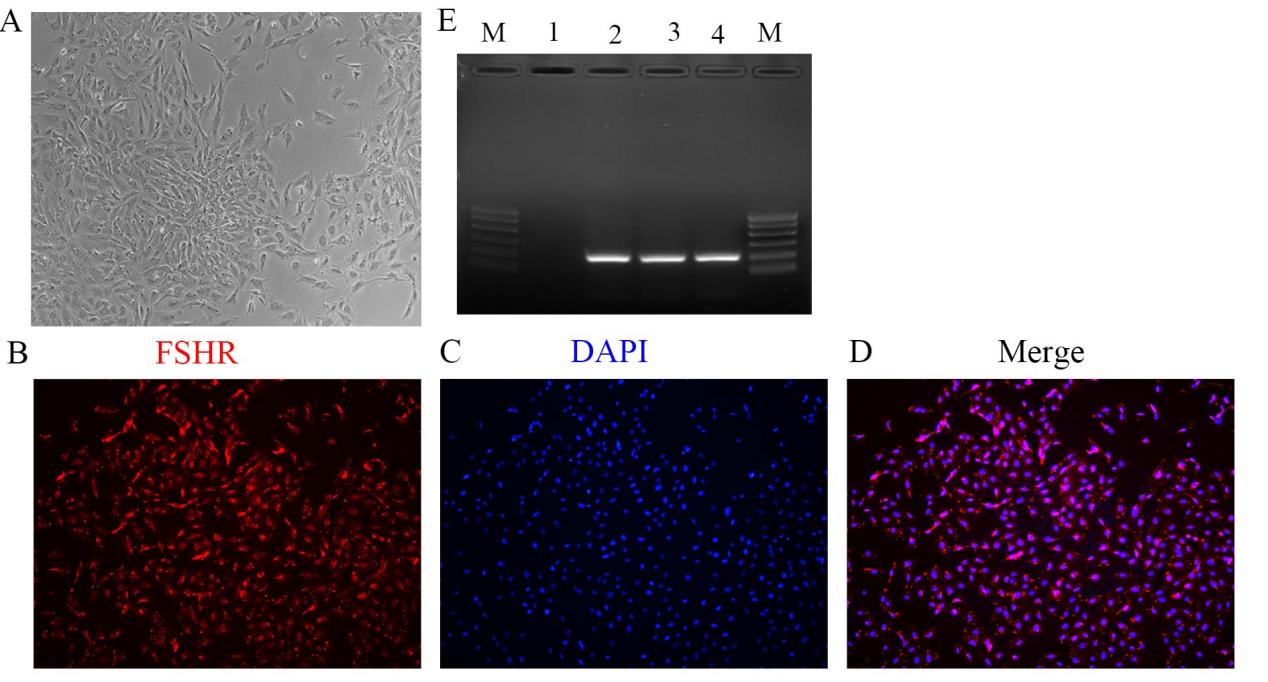


Fig.1 Isolation, culture and identification of GCs cells (A) primary culture 72h (5×); (B-D) Immunofluorescence assay (5×); (E) qPCR of FSHR M:maker 1; 1:NC; 2-4: FSHR

Expression of PAK1, CYP19A1 and PER1 in ovine gonadal axis

The differential expression of PAK1, CYP19A1 and PER1 gene in hypothalamus, pituitary, ovary, uterus and oviduct tissues of sheep was detected. As shown in the Fig.2, the PAK1 gene showed differential expression in different tissues of the gonadal axis with different abundance, and was highly expressed in the ovary and pituitary gland. And the highest expression was found relative to the hypothalamus, uterus and oviduct (P<0.01). The expression was lower in the hypothalamus. CYP19A1 gene has different degrees of expression in different tissues of gonadal axis. It was highly expressed in the ovary, and the expression level was the highest (P < 0.01). Low abundance expression in the hypothalamus (P < 0.01). The PER1 gene also showed differential expression in different tissues. It was highly expressed in pituitary and ovarian tissues, and the difference was significant (P < 0.05). Compared with other tissues, the difference was extremely significant (P < 0.01), showing low abundance expression in oviduct.


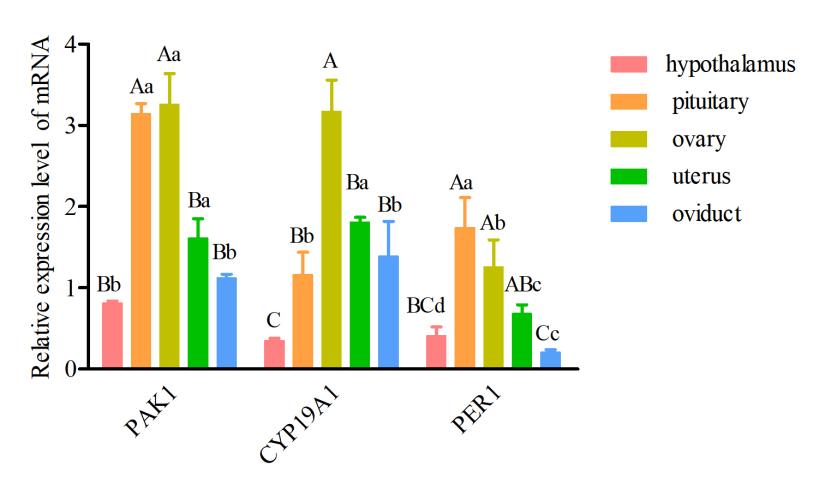


Fig.2 Tissue expression profiling of PAK1, CYP19A1, PER1 gene.

Different lowercase letters indicate significant differences (P < 0.05), and different uppercase letters indicate extremely significant differences (P < 0.01).

Detection of overexpression of PAK1, CYP19A1, PER1 gene and RNAi recombinant plasmid transfection effificiency

The overexpression vectors of PAK1, CYP19A1 and PER1 were constructed successfully (Fig.3A, B). Lipo2000 was used to transfect PAK1, CYP19A1, PER1 gene overexpression vector and siRNA interference fragment. The optimal ratio of plasmid to liposome was 1: 1.5, and the transfection effect was the best at 48 h after transfection. The cell morphology before and after transfection was better and complete. The expression of PAK1, CYP19A1, PER1 gene was detected by qPCR after transfection of overexpression recombinant plasmid and siRNA interference fragment. Compared with NC group and pEX-3 group, the expression of PAK1 in pEX-3-PAK1 group was significantly increased (P < 0.01) and increased by 78.6 times. Compared with NC group and pEX-3 group, CYP19A1 was highly expressed in pEX-3-CYP19A1 cells (P < 0.01), and was up-regulated by 77.8 times. Compared with NC group and pEX-3 group, PER1 was highly expressed in pEX-3-PER1 group (P < 0.01), which was up-regulated by 80.2 times. PAK1, CYP19A1, PER1 expression vector was successfully constructed (Fig.3C-E). Compared with the NC group, the expression of PAK1 in the si-PAK1-1 group, si-PAK1-2 group and si-PAK1-3 group was down-regulated by 74 % (P < 0.01), 54 % (P < 0.01) and 21 % (P > 0.05), respectively.Si-PAK1-1 has the best interference efficiency. According to figure B, after transfection of siRNA-CYP19A1 interference fragment, compared with NC group, the expression of CYP19A1 in si-CYP19A1-1 group, si-CYP19A1-2 group and si-CYP19A1-3 group was down-regulated by 80 % ( P < 0.01 ), 26 % ( P < 0.01 ) and 19 % ( P > 0.05 ), respectively, indicating that si-CYP19A1-1 had the best interference efficiency. According to figure C, after transfection of siRNA-PER1 interference fragment, compared with NC group, the expression of PER1 in si-PER1-1 group, si-PER1-2 group and si-PER1-3 group was down-regulated by 69 % ( P < 0.01 ), 72 % ( P < 0.01 ) and 80 % ( P > 0.05 ), respectively, indicating that si-PER1-3 had the best interference efficiency (Fig.3 F-H).


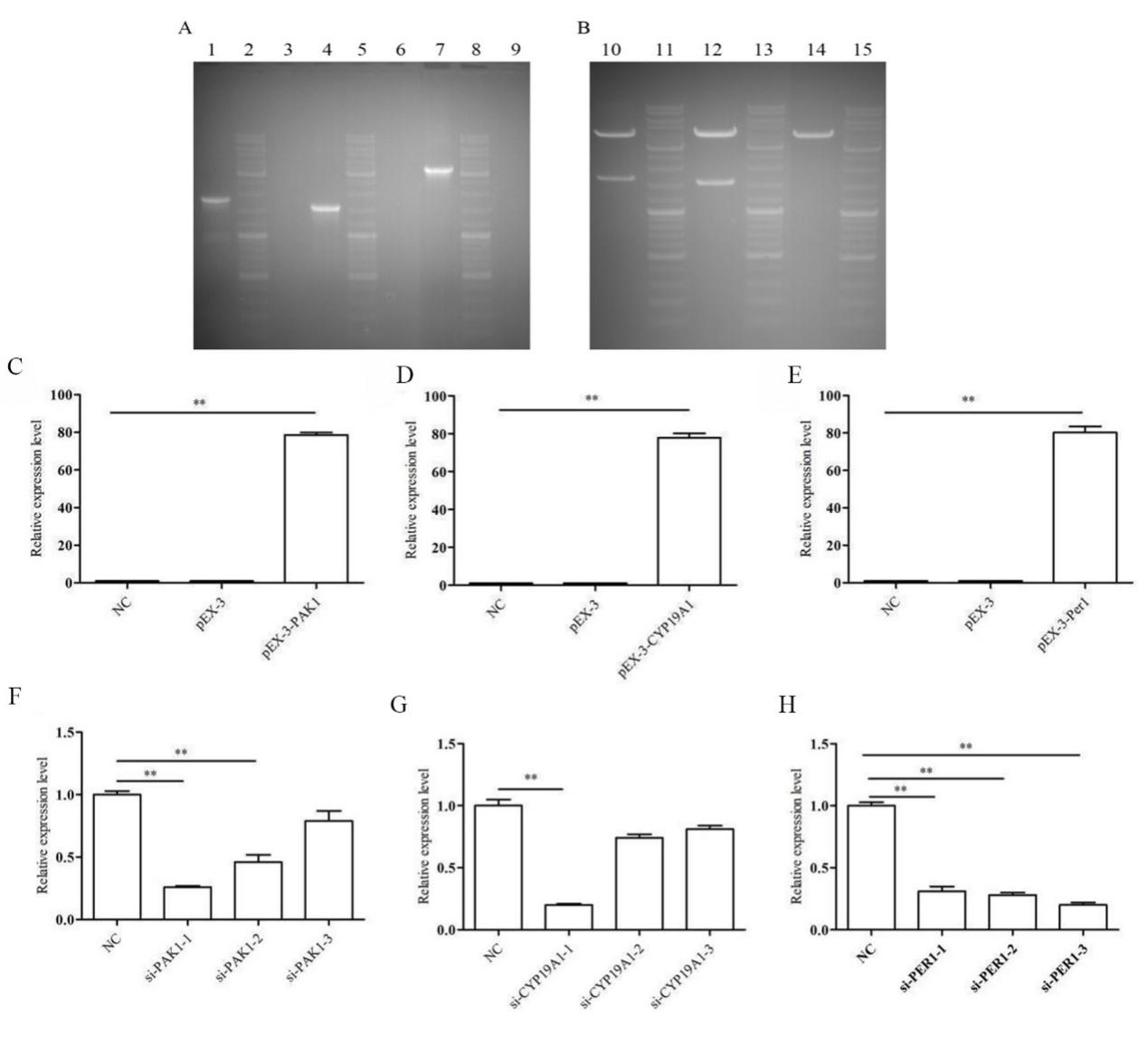


Fig.3 PCR amplification of *PAK1, CYP19A1, PER1* genes and enzyme digestion of recombination plasmids, overexpression efficiency and Interference efficiency detection of PAK1, CYP19A1 and PER1 (A) PCR amplification of *PAK1, CYP19A1, PER1* genes. (B) Enzyme digestion of pEX-3-PAK1、pEX-3-CYP19A1, pEX-3-PER1 recombination plasmids. 1: PAK1; 4: CYP19A1; 7: PER1; 3, 6, 9: NC; 2, 5, 8: DNA maker: Thermofisher SM0331; 10: Enzyme digestion of pEX-3-PAK1; 12:Enzyme digestion of pEX-3-CYP19A1; 14: Enzyme digestion of pEX-3-sheep PER1，11, 13, 15: DNA maker: Thermofisher SM0331. Overexpression efficiency of PAK1 (C), CYP19A1 (D), PER1 (E). Interference efficiency detection of PAK1 (F), CYP19A1 (G), PER1 (H)

Supplementary Figures


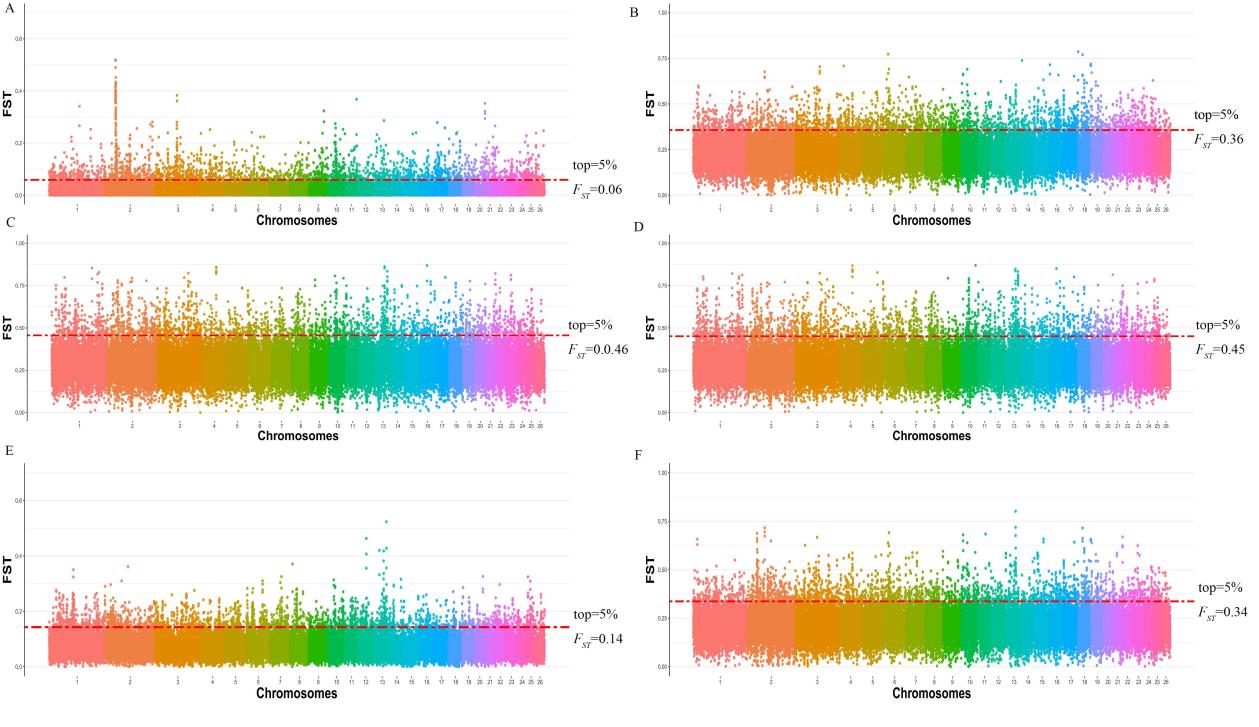


Fig.1 Top 5% manhattan map of pairwise comparison of four native sheep populations for FST values. (A) KAS versus DLS; (B) KAS versus HUS; (C) DLS versus SUF; (D) KAS versus SUF; (E) HUS versus SUF; (F) DLS versus HUS


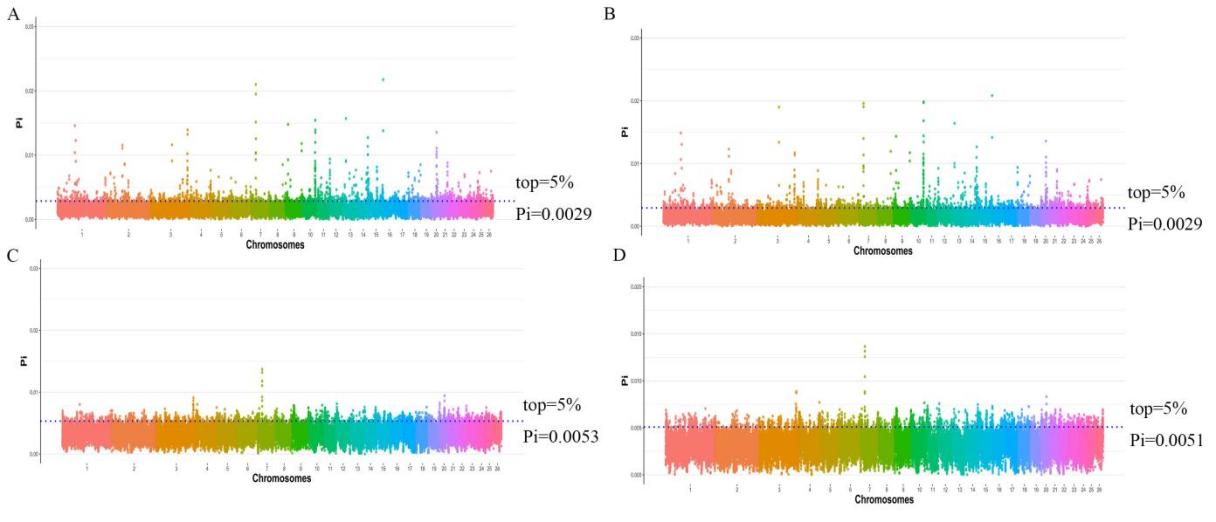


Fig.2 Top 5% manhattan map of pairwise comparison of four native sheep populations for pi ratio. (A) KAS versus DLS; (B) KAS versus HUS; (C) DLS versus SUF; (D) KAS versus SUF; (E) HUS versus SUF; (F) DLS versus HUS


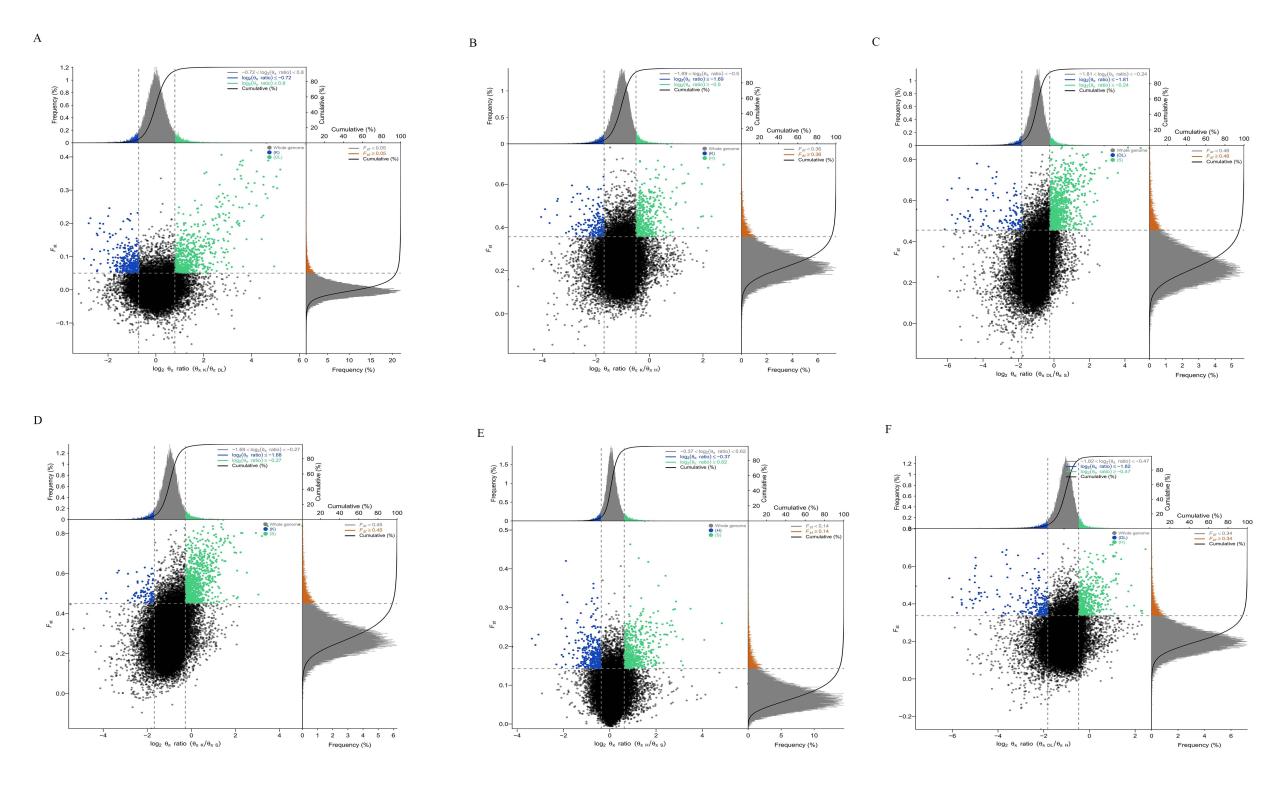


Fig.3 Selected signal of top 5% FST and top 5% ln(π ratio)/ln(2) values. (A) Kazakh sheep and Duolang sheep selected signal; (B) Kazakh sheep and Hu sheep were selected signal; (C) Selection signal of Duolang sheep and Suffolk sheep; (D) Kazakh and Suffolk sheep were selected signal; (E) Selection signal of Hu and Suffolk sheep; (F) Selection Signal of Duolang Sheep and Hu Sheep;


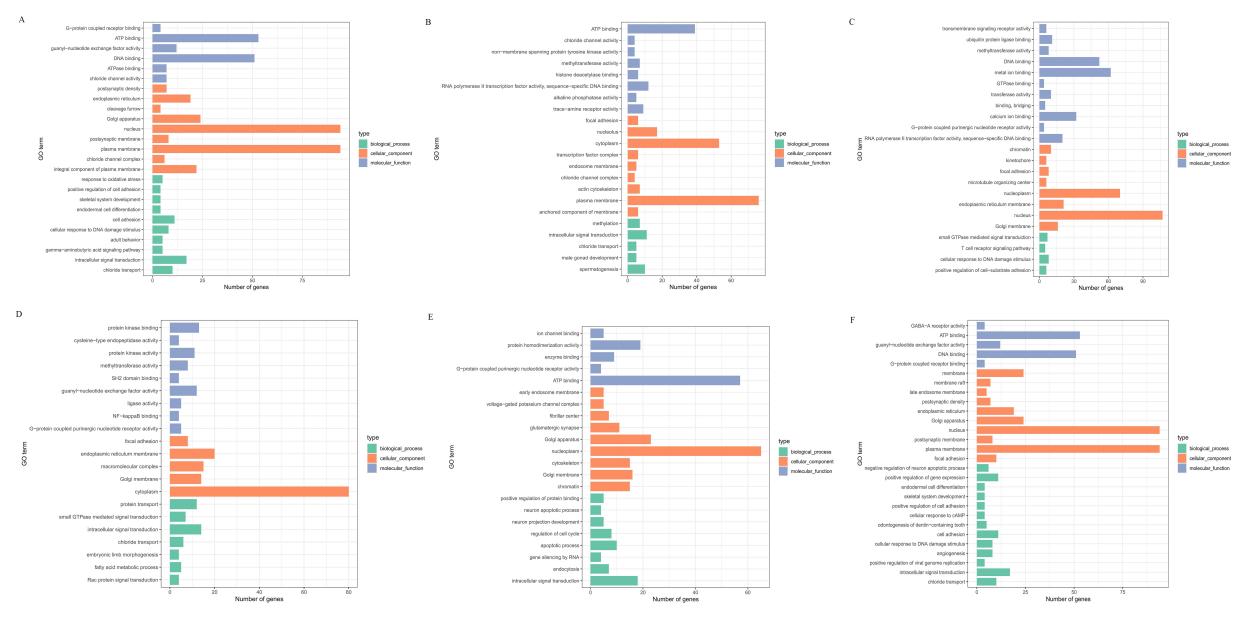


Fig.4 Go enrichment of candidate genes. (A) KAS versus DLS group; (B) KAS versus HUS group; (C) DLS versus SUF group; (D) KAS versus SUF group; (E) HUS versus SUF group; (F) DLS versus HUS group


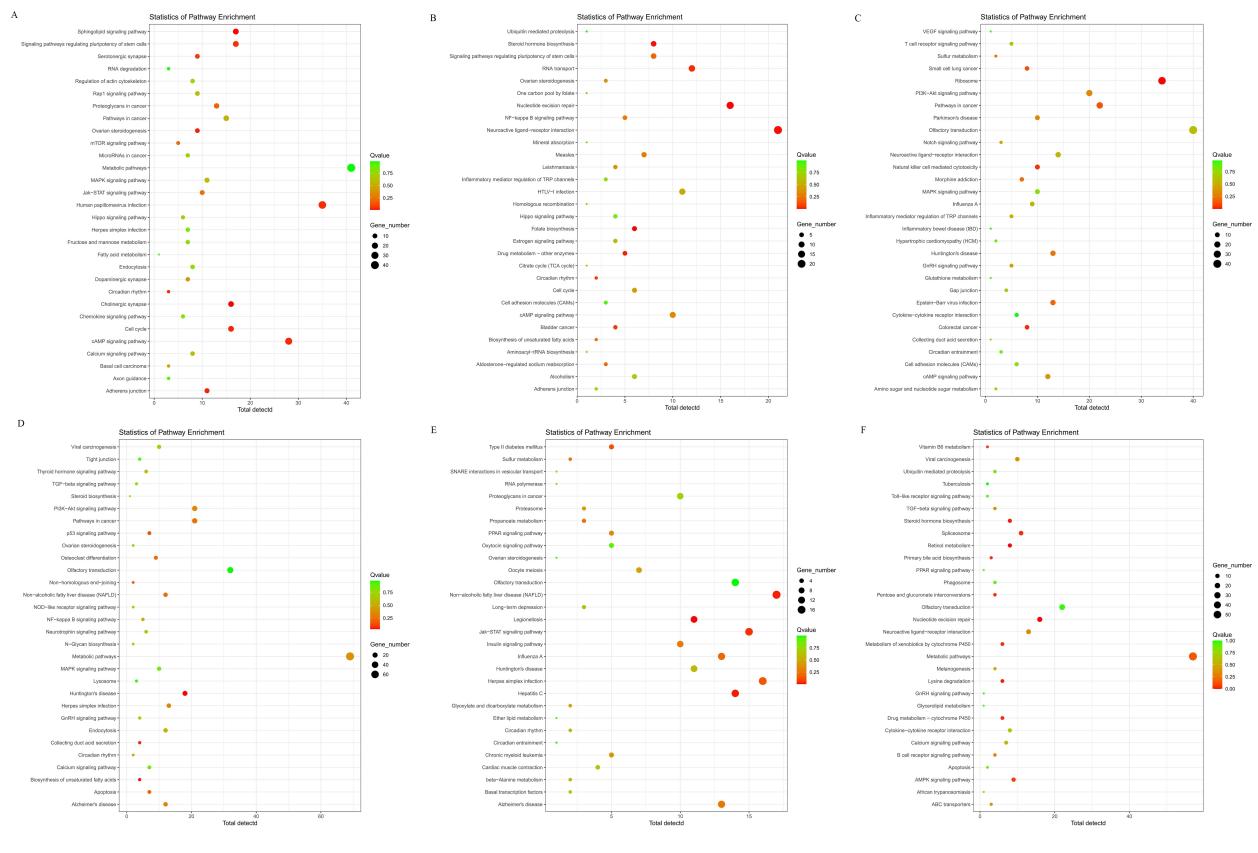


Fig. 5 KEGG pathway analysis of candidate genes. 5(A) KAS versus DLS group; (B) KAS versus HUS group; (C) DLS versus SUF group; (D) KAS versus SUF group; (E) HUS versus SUF group; (F) DLS versus HUS group

Supplementary References

1. Lin S. Screening of microRNAs related to estrus in non-breeding season and functional verification of their target genes in nutrition-induced sheep. Shihezi University, 2015.
2. Lei X. Screening and functional verification of miRNA in sheep seasonal estrus. Shihezi University, 2017.
3. Peng J. Expression regulation and function of TIMP3 gene in dairy goat ovarian granulosa cells.Northwest A & F University, 2016.
