# Supplementary figures and images for: Whole-genome resequencing of the native sheep provides insights into the microevolution and identifies genes associated with reproduction traits
Source: BMC Genomics. 2023 Jul 11;24:392. doi: 10.1186/s12864-023-09479-y (PMC10334551; doi:10.1186/s12864-023-09479-y)

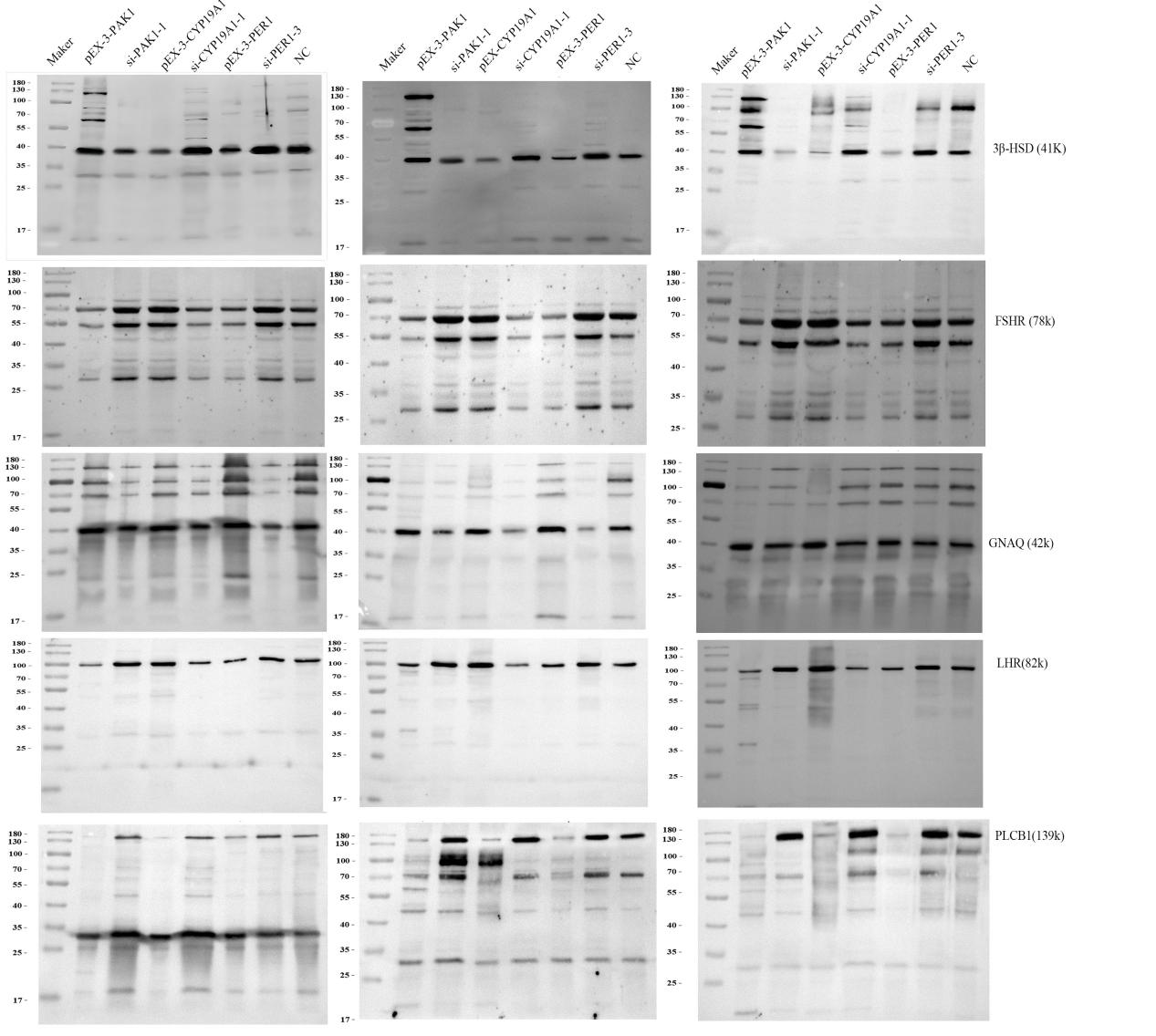

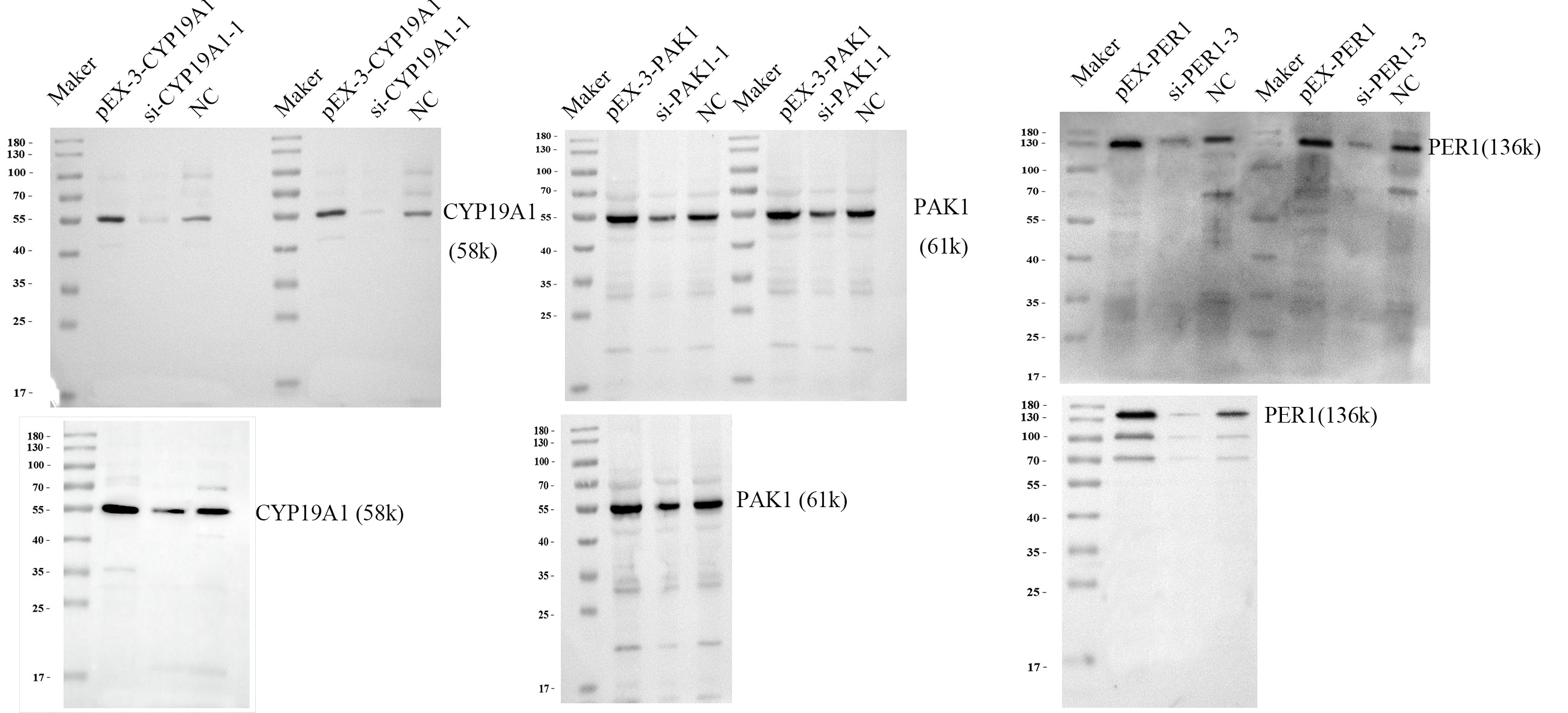

Supplement: Supplementary file 3 — Additional file 3. [file 12864_2023_9479_MOESM3_ESM.docx]
